# Precision gain versus effort with joint models using detection/non‐detection and banding data

**DOI:** 10.1002/ece3.4825

**Published:** 2019-02-05

**Authors:** Jamie S. Sanderlin, William M. Block, Brenda E. Strohmeyer, Victoria A. Saab, Joseph L. Ganey

**Affiliations:** ^1^ Rocky Mountain Research Station U.S.D.A. Forest Service Flagstaff Arizona; ^2^ Rocky Mountain Research Station U.S.D.A. Forest Service Bozeman Montana

**Keywords:** abundance, data integration, integrated population models, recruitment, study design, survival, western bluebird, wildfire effects

## Abstract

Capture–recapture techniques provide valuable information, but are often more cost‐prohibitive at large spatial and temporal scales than less‐intensive sampling techniques. Model development combining multiple data sources to leverage data source strengths and for improved parameter precision has increased, but with limited discussion on precision gain versus effort. We present a general framework for evaluating trade‐offs between precision gained and costs associated with acquiring multiple data sources, useful for designing future or new phases of current studies.We illustrated how Bayesian hierarchical joint models using detection/non‐detection and banding data can improve abundance, survival, and recruitment inference, and quantified data source costs in a northern Arizona, USA, western bluebird (*Sialia mexicana*) population. We used an 8‐year detection/non‐detection (distributed across the landscape) and banding (subset of locations within landscape) data set to estimate parameters. We constructed separate models using detection/non‐detection and banding data, and a joint model using both data types to evaluate parameter precision gain relative to effort.Joint model parameter estimates were more precise than single data model estimates, but parameter precision varied (apparent survival > abundance > recruitment). Banding provided greater apparent survival precision than detection/non‐detection data. Therefore, little precision was gained when detection/non‐detection data were added to banding data. Additional costs were minimal; however, additional spatial coverage and ability to estimate abundance and recruitment improved inference. Conversely, more precision was gained when adding banding to detection/non‐detection data at higher cost. Spatial coverage was identical, yet survival and abundance estimates were more precise. Justification of increased costs associated with additional data types depends on project objectives.We illustrate a general framework for evaluating precision gain relative to effort, applicable to joint data models with any data type combination. This framework evaluates costs and benefits from and effort levels between multiple data types, thus improving population monitoring designs.

## 1 INTRODUCTION

Robust demographic parameter estimates are critical to the conservation and management of a species (Williams, Nichols, & Conroy, 2002). Recently, increased effort has focused on developing models that combine multiple data sources for improved parameter inference (Besbeas, Freeman, Morgan, & Catchpole, 2002; Besbeas, Lebreton, & Morgan, 2003; Schaub, Gimenez, Sierro, & Arlettaz, 2007). Multiple data sources are usually combined using joint‐likelihood methods (but see Pacifici et al., 2017) and often have the advantage of increased population parameter precision (Besbeas et al., 2002; Schaub & Abadi, 2011). Several data type combinations have been used in these joint models, including capture–recapture, count, and fecundity data (integrated models; i.e., Ahrestani, Saracco, Sauer, Pardieck, & Royle, 2017; Schaub & Abadi, 2011; Wilson, Gil‐Weir, Clark, Robertson, & Bidwell, 2016), capture–recapture and census/count data (Catchpole, Freeman, Morgan, & Harris, 1998), radiotelemetry and capture–recapture data (Powell, Conroy, Hines, Nichols, & Krementz, 2000), count and detection/non‐detection data (Zipkin et al., 2017), and capture–recapture and detection/non‐detection data (Freeman & Besbeas, 2012). In addition, recent advances with Bayesian hierarchical models have illustrated the utility of integrated data models (Schaub & Abadi, 2011; Schaub et al., 2007), and created opportunities for designing future studies incorporating multiple data sources.

Despite advantages of combining multiple data sources, discussion is limited on precision gain versus effort or cost with these joint data models. Many joint model studies combine data already collected with ancillary information or information derived from existing databases (i.e., eBird [Sullivan et al., 2009, http://www.ebird.org). In these scenarios, there is little cost to incorporating additional data types, and cost‐benefit analyses may not be necessary. In field studies, however, added costs for collecting additional data types may be considerable, making it desirable to evaluate trade‐offs between precision gained from additional data and the data acquisition cost.

For example, capture–recapture techniques provide valuable information, but are often cost‐prohibitive to use at large spatial and temporal scales (landscape or regional, >5 years) (but see the Monitoring Avian Productivity and Survivorship program [MAPS]; Saracco, Royle, DeSante, & Gardner, 2010; Saracco, Royle, DeSante, & Gardner, 2012). Consequently, projects frequently employ these techniques at smaller scales to reduce effort (time and money), potentially limiting the statistical inference spatial scale (Zipkin et al., 2017; Zipkin & Saunders, 2018). Alternatively, presence–absence data for use in occupancy models (MacKenzie et al., 2002) and count data are considerably less expensive and thus can be collected at larger scales for similar cost. These data types historically provided less information than more intensive sampling approaches, but new analytical approaches now allow estimation of survival, population gains from local recruitment and immigration, and abundance using these data types with dynamic *N*‐occupancy models (Rossman et al., 2016; Zipkin et al., 2017, 2014).

Here, we illustrate how joint models using detection/non‐detection and banding data can be used to make inference on abundance at a larger spatial scale and with greater precision than was possible using single data type models. Our case study used an 8‐yr data set on western bluebird (*Sialia mexicana*) populations in ponderosa pine (*Pinus ponderosa*) forests of northern Arizona, USA. Our objectives were to: (a) estimate abundance, survival, and recruitment in a Bayesian hierarchical framework from separate models using detection/non‐detection and banding data as well as a joint model using both data types, (b) compare precision of the resulting estimates among model types, and (c) evaluate differences in precision gain versus effort (a combination of time and money) among models. This approach results in a general framework for cost‐benefit analysis that can be used to evaluate trade‐offs between precision gained from and costs associated with collecting additional data types in designing future studies.

## 2 MATERIALS AND METHODS

### 2.1 Case study

Within ponderosa pine forests in the southwestern USA, fire is a common natural disturbance (Covington & Moore, 1994; Moir, Geils, Benoit, & Scurlock, 1997) and secondary cavity‐nesting birds, like the western bluebird, rely on cavities in snags for nesting and protection from predators. Managers require information for predicting fire effects on avian community structure, especially in the Southwest where relatively little is known about avian responses (Bock & Block, 2005). Because employing capture–recapture monitoring schemes across the landscape is often cost‐prohibitive, alternative study designs and model advancements could increase inference at larger spatial and longer temporal scales.

### 2.2 Study area

Our study occurred in ponderosa pine forest within the Flagstaff Ranger District of the Coconino National Forest, northwest of Flagstaff, in north‐central Arizona (for study location maps and study area details see: Latif, Sanderlin, Saab, Block, & Dudley, 2016; Sanderlin, Block, & Strohmeyer, 2015). Two wildfires (Horseshoe Fire and Hochderffer Fire) occurred in May and June 1996 within the study area. The Horseshoe Fire encompassed ~3,500 ha and the Hochderffer fire encompassed >6,600 ha adjacent to the Horseshoe Fire. Burn severity, quantified by using the delta normalized burn ratio (dNBR; see description in “Data” section), in these areas ranged from low to high severity.

### 2.3 Field methods

#### 2.3.1 Detection/non‐detection

We sampled birds starting 1 year post‐fire using the variable‐radius point‐count method (Reynolds, Scott, & Nussbaum, 1980). Sampling occurred during the breeding season over 8 years from 1999 to 2006 (primary periods), where each season had up to three visits (secondary periods). We sampled birds at 149 points spaced at approximately 200 m intervals along 15 transects (mean = 10 points/transect, range = 3–20). Most points received three visits/year during the summer breeding season to sample bird distribution and detectability, with the exception of years 1999 and 2000 (during each of those years, we sampled one of the transects on only one occasion). Counts began within 30 min of sunrise and were completed ≤4 hr. after sunrise to sample points during periods of high bird activity. Observers remained still for 2 min after reaching a point to allow birds to resume normal activity patterns. The actual point‐count lasted 8 min.

#### 2.3.2 Banding

Artificial nest boxes were distributed randomly among approximately half of the points from each fire‐severity strata (*n* = 72 points; 27 out of 59 high severity, 25 out of 50 moderate severity, 20 out of 40 unburned) where each station had three nest boxes. After completing a point‐count (see above), observers rewalked the transect line to check nest boxes for activity and band adult birds using the boxes. Observers captured birds by target mist‐netting (Ralph, Geupel, Pyle, Martin, & DeSante, 1993), or females were removed from the nest while incubating eggs, and banded with a U.S. Geological Survey Patuxent Wildlife Research Center Bird Banding Laboratory (BBL) leg band on one leg and two BBL color bands on the other leg with a unique combination (Federal Bird Banding Permit Number 21653). Juveniles were marked with a cohort band to identify the year of birth. Resighting occurred either when (a) observers removed a female incubating eggs from the nest and quickly read her bands, or (b) observers read the color bands of perched birds in the nest vicinity. Observers attempted to recapture any juveniles resighted after their hatch year and mark them with unique BBL bands.

#### 2.3.3 Vegetation sampling

We sampled live tree basal area at all point‐count stations using a 20‐factor prism and snag basal area using a 5‐factor prism. Unburned transect stations were sampled in 1997 and 2005 and burned transect stations were sampled in 1997, 2002, and 2006.

### 2.4 Data

Detection/non‐detection data covered all points, whereas banding data were available only for nest box locations. Banding data were condensed to detections during primary periods (e.g., if an individual was detected at least once during a year, it was classified as “1” otherwise “0” for that year) due to inconsistent effort with secondary periods, which meant mark‐resight models (Arnason, Schwarz, & Gerrard, 1991) were not possible. Therefore, banding data were used in Cormack‐Jolly‐Seber models (CJS; Cormack, 1964; Jolly, 1965; Seber, 1965) instead. We used full‐identity birds only to reduce model complexity. We collapsed point‐count detections to detection/non‐detection data for use in dynamic *N*‐occupancy models (Dail & Madsen, 2011; Rossman et al., 2016), and used detections within 100 m of point‐count stations for correlating detection/non‐detection data with area quantified by fire severity (see below).

We used dNBR generated from a comparison of Landsat TM imagery recorded before and after wildfire (Eidenshink et al., 2007, http://www.mtbs.gov/) to quantify burn severity. Raw dNBR values were compiled at a 30 × 30 m resolution, and a mean dNBR was calculated for a 100‐m‐radius neighborhood centered on the point‐count station. We used the following model covariates for single data type and joint models: dNBR, an indicator for nest box location (*nbox*), time since fire (*tfire*; indicator for burned × time since fire), transect line (*TR*), snag basal area (*snag*), live tree basal area (*live*), point‐count data observer (*obs*), and sex of banded adult bird (*sex*). Transect line and observers were random effects, whereas all others were fixed effects. For numerical reasons, we used standardized covariates (mean zero and unit variance) for dNBR, snag basal area, and live tree basal area.

### 2.5 Models

**Figure 1:**
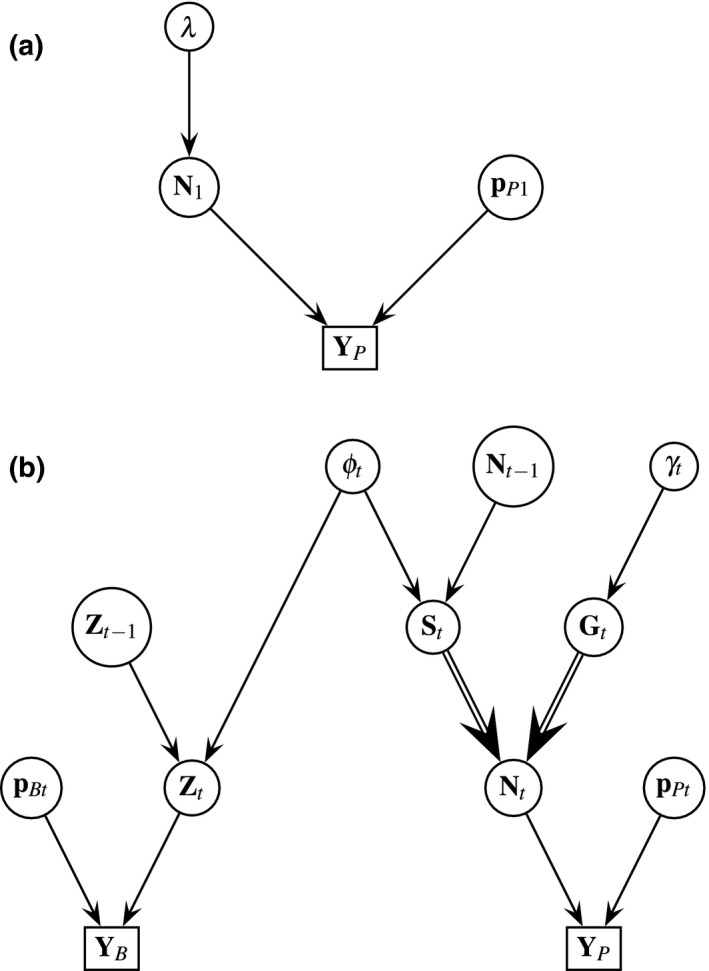
Directed acyclic graphs (DAG) of joint data model including banding (*B*) and detection/non‐detection (*P*) data for a western bluebird case study in ponderosa pine forests within Coconino National Forest in north‐central Arizona, USA between 1999 and 2006. Notation is as follows: *λ* (expected count of individuals), *N* (abundance), *ϕ* (apparent survival probability), *S* (number of individuals that survived), *γ* (recruitment), *G* (number of individuals gained through recruitment), *p*_P_ (point‐count detection probability), *p*_B_ (banding detection probability), **Z** (latent alive matrix for banded individuals), **Y**_P_ (detection/non‐detection data), and **Y**_B_ (banding data). For simplicity, case study regression coefficient parameters for *λ*, *ϕ*, *γ*, *p*_P_, and *p*_B_ were not included within the figure. Arrows indicate dependencies with parameters (circle nodes) and data (square nodes). Single arrows indicate probabilistic relationships, whereas double arrows indicate deterministic relationships. DAGs for the (a) first time period (*t* = 1) and (b) time periods after the first time period (*t* > 1) are displayed

To evaluate precision gain with the joint model (Figure 1), we first constructed separate models for each data structure and then the joint model. Results from a simulation study using the joint model indicated that our model was valid using statistical properties of accuracy, bias, percent coverage, and Bayesian credible interval (BCI) length for abundance, survival, and recruitment parameter estimates (Supporting Information Appendix S1 and S2). Because we did not know truth with our data example, we could not evaluate bias and accuracy. However, we evaluated precision of abundance, survival, and recruitment estimates (simulation study results indicated that patterns with precision were also reflected in patterns of bias and accuracy), and used the relative difference ([single model‐joint model]/joint model) in length of Bayesian credible intervals (BCIs) as our response. We were also interested in quantifying any differences between point estimates of the joint versus separate models for each data structure.

### 2.6 Detection/non‐detection data single model

We used dynamic *N*‐occupancy models (Dail & Madsen, 2011; Rossman et al., 2016) to obtain demographic estimates (abundance, survival, recruitment) from detection/non‐detection data using a state‐space modeling approach. Dynamic *N*‐occupancy models are more reliable when sites have fairly low densities (due to the reliance on detection heterogeneity to model abundance), and studies have at least 75 survey sites and 5 years of data (Rossman et al., 2016). Our study satisfied both criteria.

The state‐space model, a first‐order Markov process, describes two‐time series, the biological state process and the observation process, that run in parallel and incorporate both process and sampling error in the same framework (i.e., Buckland, Newman, Thomas, & Koesters, 2004). We used a detection/non‐detection data matrix **Y**_P_, where element *Y_Pjkt_* was a binary indicator of species detection. When *Y_Pjkt_* = 1, a western bluebird was detected at point *j *(*j = *1,…, 149) during session *k* (*k = *1, 2, 3) of year *t* (*t = *1,…, 8). Changes in abundance *N*_jt_ over time were a function of the biological state processes. We modeled abundance at each site *j* during the first year of sampling (*t* = 1) using site‐level covariates to describe the expected value of *λ*_j_ of a Poisson distribution (Equation 1):

(1) $$\left[N_{j1}|\lambda_{j}\right]∼\mathrm{Poisson}\left(\lambda_{j}\right),$$

where expected count (Equation 2) *λ*_j_ was:

(2) $$\log\left(\lambda_{j}\right)=a_{0}+a_{1}\times dNBR_{j}+a_{2}\times nbox_{j}+a_{3}\times {\mathrm{tfire}}_{1}+a_{4R_{j}},$$

and each point *j* was located within a transect *R* (*R = *1, …, 15), indexed by *R_j_*. Parameter *a*_4_ *_R_* was a normal random effect for transect *R* with mean 0 and a uniform (0, 5) prior on *σ* (e.g., *a*_4_ *_R_* ~ Normal (0, *σ* ^2^)). Parameters *a*_0_, *a*_1_, *a*_2_, and *a*_3_ had normal (*µ* **= **0, *σ* ^2^ = 0.1) priors.

Abundance at *t* > 1 was a function of the number of individuals that survived (*S_jt_*) and the number of individuals that were recruited (*G_jt_*) from *t*−1 to *t*: $N_{\mathrm{jt}}=S_{\mathrm{jt}}+G_{\mathrm{jt}}$. We modeled *S*_jt_ (Equation 3) as:

(3) $$\left[S_{\mathrm{jt}}|N_{jt-1},\varphi_{t}\right]∼Bin\left(N_{jt-1},\varphi_{t}\right),$$

where apparent annual survival probability (Equation 4) from time *t*−1 to *t*, *φ_jt_*, was:

(4) $$\mathrm{logit}\left(\varphi_{\mathrm{jt}}\right)=b_{0}+b_{1}\times nbox_{j}+b_{2}\times {\mathrm{tfire}}_{t}+b_{3}\times live_{\mathrm{jt}}+b_{4}\times snag_{\mathrm{jt}}.$$

Parameters *b*_1_, *b*_2_, *b*_3_, and *b*_4_ had normal (*µ* **= **0, *σ* ^2^ = 0.1) priors, while *b*_0_ had a normal (*µ* **= **0, *σ* ^2^ = 1) prior. We modeled *G_jt_* (Equation 5) using a Poisson distribution:

(5) $$\left[G_{\mathrm{jt}}|\gamma_{\mathrm{jt}}\right]∼\mathrm{Poisson}\left(\gamma_{\mathrm{jt}}\right),$$

where the expected number of individuals gained (Equation 6) to site *j* between *t* −1 and *t* was:

(6) $$\log\left(\gamma_{\mathrm{jt}}\right)=c_{0}+c_{1}\times nbox_{j}+c_{2}\times {\mathrm{tfire}}_{t}+c_{3}\times live_{\mathrm{jt}}+c_{4}\times snag_{\mathrm{jt}}.$$

Parameters *c*_0_, *c*_1_, *c*_2_, *c*_3_, and *c*_4_ had normal (*µ* **= **0, *σ* ^2^ = 0.1) priors. This recruitment estimate included births and immigration, but empirical data suggested immigration was negligible (see next section).

We modeled the observation process (Royle & Nichols, 2003) (Equation 7) as:

(7) $$\left[Y_{\mathrm{Pjkt}}|N_{\mathrm{jt}},p_{\mathrm{Pjkt}}\right]∼\mathrm{Bern}\left(1-{\left(1-p_{\mathrm{Pjkt}}\right)}^{N_{\mathrm{jt}}}\right),$$

where detection probability (Equation 8) was:

(8) $$\mathrm{logit}\left(p_{\mathrm{Pjkt}}\right)=d_{0}+d_{1}\times nbox_{j}+d_{2o_{\mathrm{jkt}}},$$

and each point *j, *session *k*, year *t* had observer *o *(*o = *1, …, 9), indexed by *o_jkt_*. Parameter *d*_2_ *_o_* was a normal random effect for observer *o* with mean 0 and a uniform (0, 5) prior on *σ* (e.g., *d*_2_ *_o_* ~ Normal (0, *σ* ^2^)). We assumed there was no individual heterogeneity with detection probability. Priors for parameters *d*_0 _and *d*_1_ were normal (*µ* **= **0, *σ* ^2^ = 1) and normal (*µ* **= **0, *σ* ^2^ = 0.1), respectively.

### 2.7 Banding data single model

We used the CJS model (Cormack, 1964; Jolly, 1965; Seber, 1965) in a state‐space approach (Royle & Dorazio, 2008) to obtain survival estimates from banding data. Western bluebirds had high site‐fidelity in this study between years. Only 3 out of 471 (0.6%) individuals detected were detected at two different transects between years, and 16 out of 471 (3.4%) individuals were detected at different points within a transect between years. Thus, to reduce model complexity we assumed no movement between nest box locations between primary periods. The model could be expanded to include movement between points, however (i.e., Hestbeck, Nichols, & Malecki, 1991).

We used a latent alive matrix **Z** of dimension *N_B_* × *t* of *N_B_* individuals ever captured during banding within the study period spanning *t* sampling years, where element *Z_ijt_* took the value “1” if individual *i* at point *j* was alive and encountered during the time interval between sample *t*−1 and *t* and “0” otherwise. The CJS model is conditional on time of first capture of each individual, *f_i_*. We modeled initial state (Equation 9) for individual *i* as a Bernoulli trial with apparent survival probability *φ_jt_*, where $t=f_{i}+1,\ldots ,7$:

(9) $$\left[Z_{\mathrm{ijt}}|Z_{ijt-1},\varphi_{\mathrm{jt}}\right]∼\mathrm{Bern}\left(Z_{ijt-1}\times \varphi_{\mathrm{jt}}\right),$$

where apparent survival probability was modeled the same as Equation 4.

We modeled the observation process (Equation 10) conditional on the true process with banding data of individual *i*, *Y*_B_ *_ijt_*, using a Bernoulli trial:

(10) $$\left[Y_{\mathrm{Bijt}}|Z_{\mathrm{ijt}},p_{\mathrm{Bi}}\right]∼\mathrm{Bern}\left(Z_{\mathrm{ijt}}\times p_{\mathrm{Bi}}\right),$$

where individual detection probability (Equation 11) was:

(11) $$\mathrm{logit}\left(p_{\mathrm{Bi}}\right)=e_{0}+e_{1}\times sex_{i}.$$

The prior for intercept *e*_0 _was uniform (0, 1) and for *e*_1 _was normal (*µ* **= **0, *σ* ^2^ = 0.1).

### 2.8 Joint model

Because we assumed these data structures were independent (see Supporting Information Appendix S3 for exploration of independence assumption), we factored the following components (Equation 12) of the joint posterior distribution, also depicted in the directed acyclic graph (DAG) (Figure 1):

(12) $$\begin{array}{c} \left[\lambda ,N,p_{P},S,G,\gamma ,\phi ,Z,p_{B}|Y_{B},Y_{P}\right]\propto \left[Y_{P1}|N_{1},p_{P1}\right]\times \left[N_{1}|\lambda \right]\times \\ \left[Y_{B}|p_{B},Z_{t}\right]\times \left[Z_{t}|Z_{t-1},\phi_{t}\right]\times \left[S_{t}|N_{t-1},\phi_{t}\right]\times \left[N_{t}|S_{t},G_{t}\right]\times \\ \left[G_{t}|\gamma_{t}\right]\times \left[Y_{P}|p_{\mathrm{Pt}},N_{t}\right]\times \left[\lambda ,p_{P},\gamma ,{\phi ,p}_{B}\right] \end{array}$$

For simplicity, case study regression coefficient parameters for *λ*, *ϕ*, *γ*, *p*_P_, and *p*_B_ were not included within Equation 12.

### 2.9 Inference

We conducted model selection for nested models using indicator variable selection (O'Hara & Sillanpää, 2009). Individual coefficients for our predictor variables **β** were modified with a binary indicator variable *v*_i_, such that $\beta_{i}=v_{i}\times \theta_{i}$ where *θ_i_* was the original parameter. For each single data type and joint model, we evaluated all coefficients for our predictor variables at the same time. All indicator variables *v*_i_ had Bernoulli (0.5) priors. If the posterior mean for *v*_i_ was closer to one than zero, the covariate had more model support than if *v*_i_ was closer to zero. We defined strong model support as the posterior mean > 0.5. We implemented these Bayesian hierarchical models (Gelman, Carlin, Stern, & Rubin, 2004) in JAGS (Plummer, 2003) using the *rjags* package in R (R Core Team, 2017) (see Supporting Information Appendix S4 for model code). We also used package *jagsUI* (Kellner, 2016) a wrapper around *rjags* to implement parallel processing, and package *coda* (Plummer, Best, Cowles, & Vines, 2006) to obtain posterior parameter estimates. We ran 3 parallel chains for single and joint models (joint data model: total length 500,000 *iterations *[*it*], burn‐in 400,000 *it*, thinning 10 *it*; detection/non‐detection data only model: total length 800,000 *it*, burn‐in 700,000 *it*, thinning 10 *it*; banding data only model: total length 400,000 *it*, burn‐in 300,000 *it*, thinning 10 *it*) to estimate the posterior distribution median of model parameters and 95% Bayesian Credible Intervals (BCI). Convergence was reached ($\hat{R}$ < 1.1 [Brooks & Gelman, 1998]). We assessed goodness‐of‐fit (GOF) using the squared loss statistic for a Bayesian *p*‐value (Gelman et al., 2004:162).

### 2.10 Effort

We used estimated project costs for detection/non‐detection and banding data to quantify effort, which was a combination of time and money. We evaluated effort with single and combined data sources. To illustrate the types of costs that might be included within a study to evaluate effort and precision, we quantify costs here with data types specific to our study. We note that cost functions are often study‐specific, but general cost categories exist with establishment, sampling unit, sampling occasion (and combinations of sampling unit by sampling occasion) components. We do not include costs associated with collecting covariate information, since relative costs would be the same in our case study for banding data and detection/non‐detection data only models (e.g., we would include the same vegetation data in single data models). However, field costs may be substantial for collecting covariate data and differ between data types in other studies, so this would be important to include in such cases. We used the following cost function (*C_p_*) for detection/non‐detection data (Equation 13):

(13) $$C_{P}=C_{0,P}+C_{1,P}\times s+C_{2,P}\times k\times s\times t+C_{3,P}\times k\times t,$$

where *s* was the number of sampling units (*s* = 149 points), *k* was the number of sampling occasions (*k* = 3), *t* was the number of years (*t* = 8), *C*_0,_ *_P_* was the initial project startup cost with detection/non‐detection data which included study design and equipment costs, *C*_1,_ *_P_* was the additional establishment cost per sampling unit for detection/non‐detection data, *C*_2,_ *_P_* was the additional cost to sample each sampling unit per sampling occasion per year for detection/non‐detection data, and *C*_3,_ *_P_* was the additional cost per sampling occasion per year for detection/non‐detection data. For our example, we used the following cost estimates: *C*_0_ *_,p_* = $21,900, *C*_1,_ *_p_* = $19 (per point cost includes two observers’ salaries and equipment costs), *C*_2,_ *_p_* = $12 (per point/occasion/year cost includes two observers’ salaries), and *C*_3,_ *_p_* = $64 (per occasion per year cost for data entry with two observers). Based on our field study, we assumed that two observers (one biological technician, one crew leader) could sample or establish 20 points total per day (10 points per observer).

We used the following cost function (*C*_B_) for banding data (Equation 14):

(14) $$C_{B}=C_{0,B}+C_{4,B}\times s+C_{5,B}\times s\times t+C_{6,B}\times t,$$

where *s* was the number of sampling units (*s* = 216 boxes, 72 points with 3 boxes per point), *t* was the number of years (*t* = 8), *C*_0,_ *_B_* was the initial project startup cost which included study design and equipment costs for banding data, *C*_4,_ *_B_* was the additional establishment cost per sampling unit for banding data, *C*_5,_ *_B_* was the additional cost to sample each sampling unit per year for banding data, and *C*_6,_ *_B_* was the additional cost to sample per year (without respect to number of sampling units) for banding data. For our example, we used the following cost estimates: *C*_0,_ *_B_* = $23,400, *C*_4,_ *_B_* = $105 (per nest box cost includes two observers’ salaries and equipment costs), *C*_5,_ *_B_* = $55 (per nest box per year cost includes two observers’ salaries), and *C*_6,_ *_B_* = $264 (data entry and equipment costs per year). Based on our field study, we assumed that two observers (one biological technician, one crew leader) could sample or establish one box per hour.

We used the following cost function (*C_J_*) for the joint data structures (Equation 15) of detection/non‐detection and banding data:

(15) $$C_{j}=C_{0,J}+C_{1,J}\times s+C_{2,J}\times k\times s\times t+C_{3,J}\times k\times t+C_{4,J}\times s+C_{5,J}\times s\times t+C_{6,J}\times t,$$

where *s* was the number of sampling units (*s* = 77 points for the *C*_1,_ *_J _*term, *s* = 149 points for the *C*_2,_ *_J _*term, *s* = 216 boxes for the *C*_4,_ *_J _*and *C*_5,_ *_J _*terms), whereas *k*, *t*, *C*_0,_ *_J_*, *C*_1,_ *_J_*, *C*_2,_ *_J_*, *C*_3,_ *_J_*, *C*_4,_ *_J_*, *C*_5,_ *_J_*, and *C*_6,_ *_J _*were the same as above, but for both detection/non‐detection and banding data sources. For our example, we used the following cost estimates (note that cost estimates were not the same as above due to differing amounts of time allocated for sampling and how travel time was distributed between sampling methods): *C*_0,_ *_J_* = $28,400, *C*_1,_ *_J_* = $19 (per point cost includes two observers’ salaries and equipment costs), *C*_2,_ *_J_* = $10 (per point/occasion/year cost includes two observers’ salaries), *C*_3,_ *_J_* = $85 (per occasion per year cost for data entry with two observers), *C*_4,_ *_J_* = $105 (per nest box cost includes two observers’ salaries and equipment costs), *C*_5,_ *_J_* = $46 (per nest box per year cost includes two observers’ salaries), and *C*_6,_ *_J_* = $200 (equipment costs per year). Based on our field study, we assumed that two observers (one biological technician, one crew leader) could sample or establish 20 points total per day (10 points per observer) and sample or establish one box per hour.

## 3 RESULTS

### 3.1 Data summary

**Figure 2:**
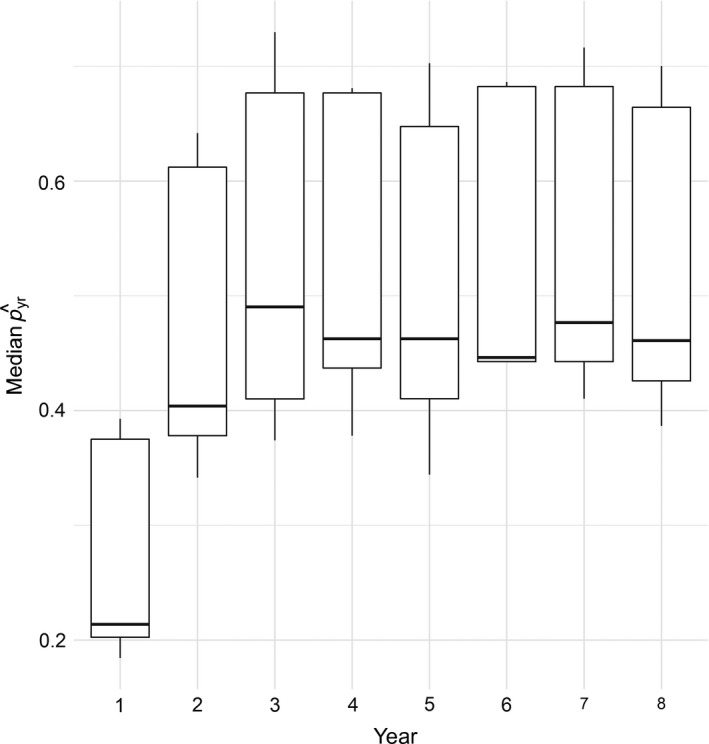
Box and whisker plot for detection probability medians of all sampling locations by year for detection/non‐detection data from the joint data model from a western bluebird case study in ponderosa pine forests within Coconino National Forest in north‐central Arizona, USA between 1999 and 2006. Detection probability estimates from detection/non‐detection data were originally for each secondary period but converted to primary periods (e.g., ${\hat{p}}_{\mathrm{yr}}=1-\left[\prod_{i=1}^{3}\left(1-{\hat{p}}_{\mathrm{session}\;i}\right)\right]$)

We banded 471 adult western bluebirds over 8 years. Number of points where *y_jkt_* = 1 for western bluebirds across point counts was comparable across sessions during each year (session 1 [68, 65, 65, 71, 65, 88, 89, 96], session 2: [76, 79, 60, 63, 67, 81, 113, 85], session 3: [77, 87, 57, 68, 73, 71, 88, 76]). There was no evidence of model overdispersion with single data type models and moderate overdispersion with the joint model (Bayesian *p*‐values: detection/non‐detection data only model 0.504, band data only model 0.478, joint data model 0.294). Detection probabilities for banding and detection/non‐detection data were relatively high (banding data posterior medians were 0.480 males and 0.700 for females; posterior median range for yearly detection probability with detection/non‐detection data [0.185–730]) with the exception of detection/non‐detection data in year 1 (Figure 2).

### 3.2 Estimates of abundance, survival, and recruitment

Model support for individual covariates varied among model types (Tables 1 and 2). Relationships reported as positive or negative were statistically significant (e.g., 95% credible intervals [CI] did not include zero). For the detection/non‐detection data model, there was strong model support (mean posterior of binary indicator variable >0.5 from indicator variable selection [O'Hara & Sillanpää, 2009]) for initial abundance with the covariate transect (negative and positive posterior medians for individual transects, but 95% CIs included zero). For the detection/non‐detection data model apparent survival probability, nest box indicator (negative posterior median, but 95% CI included zero), time since fire (positive posterior median, but 95% CI included zero), and snag basal area (negative relationship) had strong model support. Snag basal area (positive relationship) had strong model support for recruitment with the detection/non‐detection data model. For the detection/non‐detection data model, the following covariates nest box indicator (positive relationship) and observer (negative relationship) had strong model support for point‐count detection probability. None of the covariates had model support with the banding data model.

**Table 1:** Mean estimates of posterior support using indicator variable selection for model covariates in joint data and single data models with detection/non‐detection and banding data from a western bluebird case study in ponderosa pine forests within Coconino National Forest in north‐central Arizona, USA between 1999 and 2006

Parameter	Covariate	Detection/non‐detection data only model	Banding data only model	Joint data model
*N*	*a*_1_ (*dnbr*)	0.032	NA	0.042
	*a*_2_ (*nbox*)	0.101	NA	0.070
	*a*_3_ (*tfire*)	0.339	NA	0.763*
	*a*_4_ (*transect*)	0.908*	NA	0.522*
*ϕ*	*b*_1_ (*nbox*)	0.924*	NA	0.089
	*b*_2_ (*tfire*)	0.955*	0.013	0.021
	*b*_3_ (*live*)	0.377	0.153	0.281
	*b*_4_ (*snag*)	1.000*	0.164	0.703*
*γ*	*c*_1_ (*nbox*)	0.091	NA	0.084
	*c*_2_ (*tfire*)	0.060	NA	1.000*
	*c*_3_ (*live*)	0.108	NA	0.517*
	*c*_4_ (*snag*)	1.000*	NA	0.577*
$p_{P}$	*d*_1_ (*nbox*)	1.000*	NA	1.000*
	*d*_2_ (*obs*)	1.000*	NA	1.000*
$p_{B}$	*e*_1_ (*sex*)	NA	0.318	0.962*

Note

Estimates marked with an “*” indicate strong model support (posterior mean > 0.5). “NA” indicated the parameter was not part of the model. Data sources included detection/non‐detection and banding data. Parameters included *N* (abundance), *ϕ *(apparent survival probability), *γ *(recruitment), *p*_P_ (point‐count detection probability), *p*_B_ (banding detection probability).

**Table 2:** Median estimates (95% credible intervals) for model covariates in joint data and single data models with detection/non‐detection and banding data from a western bluebird case study in ponderosa pine forests within Coconino National Forest in north‐central Arizona, USA between 1999 and 2006

Parameter	Covariate	Covariate subcategories	Detection/non‐detection data only model	Banding data only model	Joint data model
*N*	*a*_3_ (*tfire*)		—	NA	0.206 (0.000, 0.356)
	*a*_4_ (*transect*)	*transect A*	−0.087 (−0.548, 0.298)	NA	0.000 (−0.442, 0.284)
		*transect B*	0.248 (−0.111, 0.673)	NA	0.000 (−0.127, 0.566)
		*transect C*	0.359 (−0.044, 0.858)	NA	0.000 (−0.011, 0.837)
		*transect D*	−0.143 (−0.783, 0.398)	NA	0.000 (−0.656, 0.361)
		*transect E*	−0.063 (−0.654, 0.480)	NA	0.000 (−0.557, 0.423)
		*transect F*	0.140 (−0.443, 0.824)	NA	0.000 (−0.383, 0.651)
		*transect G*	0.304 (−0.235, 0.977)	NA	0.000 (−0.243, 0.782)
		*transect H*	0.000 (−0.400, 0.429)	NA	0.000 (−0.319, 0.405)
		*transect I*	−0.396 (−1.009, 0.031)	NA	0.000 (−0.869, 0.013)
		*transect J*	0.000 (−0.514, 0.586)	NA	0.000 (−0.458, 0.506)
		*transect K*	−0.591 (−1.283, 0.006)	NA	0.000 (−1.133, 0.032)
		*transect N*	−0.124 (−0.801, 0.369)	NA	0.000 (−0.623, 0.336)
		*transect R*	0.000 (−0.512, 0.489)	NA	0.000 (−0.427, 0.428)
		*transect X*	0.536 (−0.017, 1.189)	NA	0.000 (−0.084, 1.008)
		*transect Z*	−0.072 (−0.617, 0.367)	NA	0.000 (−0.540, 0.280)
*ϕ*	*b*_1_ (*nbox*)		−0.877 (−1.521, 0.000)	NA	—
	*b*_2_ (*tfire*)		0.219 (0.000, 0.294)	—	—
	*b*_4_ (*snag*)		−5.426 (−8.762, −3.140)	—	−1.067 (−2.437, 0.000)
*γ*	*c*_2_ (*tfire*)		—	NA	0.092 (0.064, 0.120)
	*c*_3_ (*live*)		—	NA	0.098 (0.000, 0.517)
	*c*_4_ (*snag*)		0.581 (0.366, 0.774)	NA	0.230 (−0.460, 0.706)
p_p_	*d*_1_ (*nbox*)		1.134 (0.750, 1.620)	NA	0.772 (0.578, 0.978)
	*d*_2_ (*obs*)	*observer 1*	−2.328 (−2.999, −1.783)	NA	−1.799 (−2.188, −1.473)
		*observer 2*	−2.637 (−3.313, −2.088)	NA	−2.140 (−2.553, −1.792)
		*observer 3*	−2.558 (−3.266, −1.931)	NA	−1.961 (−2.429, −1.527)
		*observer 4*	−2.340 (−3.023, −1.781)	NA	−1.828 (−2.260, −1.437)
		*observer 5*	−2.047 (−2.734, −1.464)	NA	−1.503 (−1.919, −1.137)
		*observer 6*	−2.278 (−2.953, −1.741)	NA	−1.758 (−2.142, −1.438)
		*observer 7*	−2.521 (−3.214, −1.914)	NA	−1.959 (−2.418, −1.546)
		*observer 8*	−2.001 (−2.673, −1.441)	NA	−1.455 (−1.849, −1.110)
		*observer 9*	−2.319 (−3.767, −0.851)	NA	−1.823 (−3.179, −0.422)
p_B_	*e*_1_ (*sex*)		NA	—	0.956 (0.000, 1.717)

Note

We include all covariates that had strong model support with indicator variable selection (posterior mean > 0.50). “NA” indicated the parameter was not part of the model and “—” indicated that the covariate did not have strong model support. Parameters included *N* (abundance), *ϕ *(apparent survival probability), *γ *(recruitment), *p*_P_ (point‐count detection probability).

For the joint data model, time since fire (positive posterior median, but 95% CI included zero) and transect (negative and positive posterior medians for individual transects, but 95% CIs included zero) had strong support for initial abundance. Apparent survival probability had strong model support with snag basal area (negative posterior median, but 95% CI included zero). Recruitment had strong model support with covariates time since fire (positive relationship), live tree basal area (positive posterior median, but 95% CI included zero), and snag basal area (positive posterior median, but 95% CI included zero). Point‐count detection probability had model support with covariates nest box indicator (positive relationship), and observer (negative posterior medians, but 95% CIs included zero).

**Figure 3:**
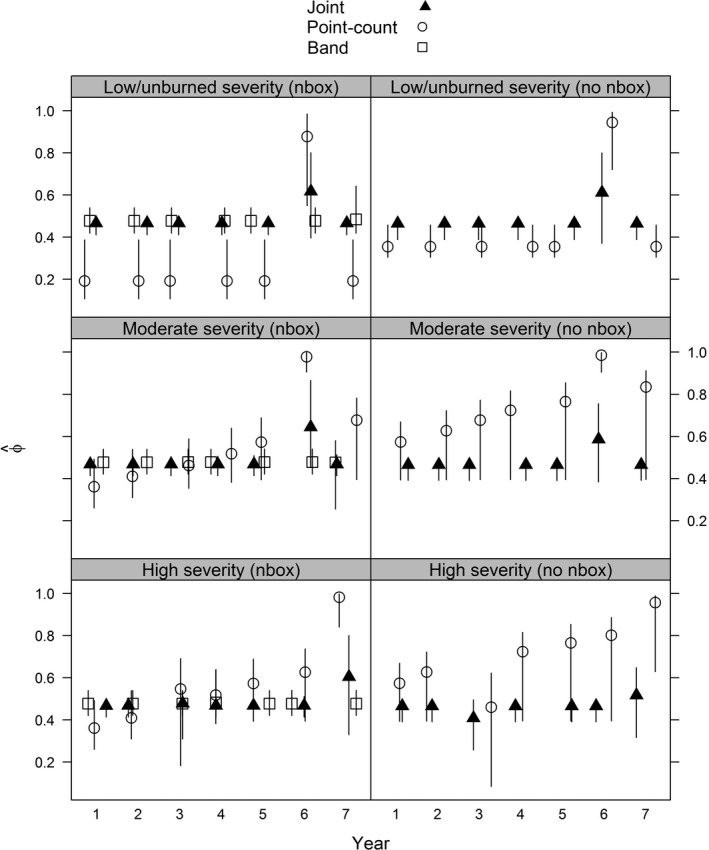
Apparent survival (*φ*) posterior median estimates and associated Bayesian credible intervals from a western bluebird case study in ponderosa pine forests within Coconino National Forest in north‐central Arizona, USA between 1999 and 2006. Estimates were derived using data from a single bird point‐count station and models using detection/non‐detection data only, banding data only, and a joint model using both data types. Example sampling locations included those with (*nbox*) and without nest boxes (*no nbox*) for high (Transect A, points 2 and 6), moderate (Transect B, points 2 and 1), and low/unburned (Transect J, points 6 and 1) burn severity

**Figure 4:**
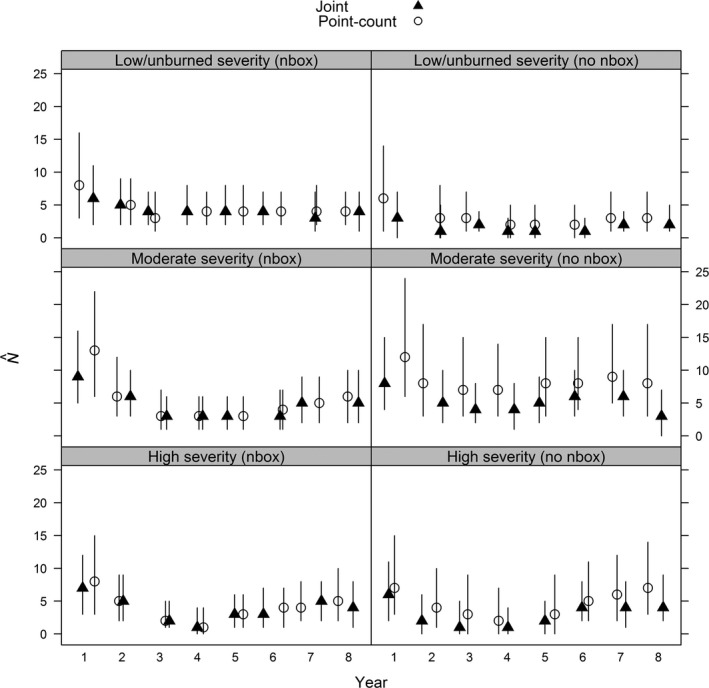
Abundance (*N*) posterior median estimates and associated Bayesian credible intervals from a western bluebird case study in ponderosa pine forests within Coconino National Forest in north‐central Arizona, USA between 1999 and 2006. Estimates were derived using data from a single bird point‐count station and models using detection/non‐detection data only, banding data only, and a joint model using both data types. Example sampling locations included those with (*nbox*) and without nest boxes (*no nbox*) for high (Transect A, points 2 and 6), moderate (Transect B, points 2 and 1), and low/unburned (Transect J, points 6 and 1) burn severity

**Figure 5:**
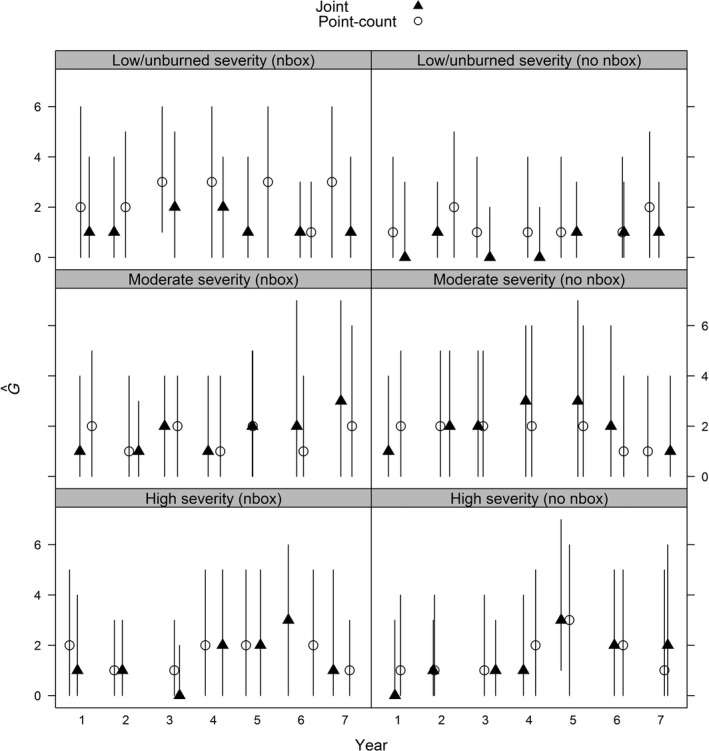
Recruitment (*G*) posterior median estimates and associated Bayesian credible intervals from a western bluebird case study in ponderosa pine forests within Coconino National Forest in north‐central Arizona, USA between 1999 and 2006. Estimates were derived using data from a single bird point‐count station and models using detection/non‐detection data only, banding data only, and a joint model using both data types. Example sampling locations included those with (*nbox*) and without nest boxes (*no nbox*) for high (Transect A, points 2 and 6), moderate (Transect B, points 2 and 1), and low/unburned (Transect J, points 6 and 1) burn severity

To illustrate how precision of estimates varied among models, we used examples from two points (one with and one without a nest box) from three transects of high, moderate, and low/unburned fire severity. For apparent survival probability, both the joint and band data only models had relatively constant survival over time, whereas survival with the detection/non‐detection data only model increased slightly with increased time since fire with points at high and moderate severity (Figure 3). Precision increase was greatest for survival estimates, followed by abundance, especially with locations that did not have nest boxes, and minimal increases in precision with recruitment (Figures 4 and 5).

### 3.3 Effort comparison

**Figure 6:**
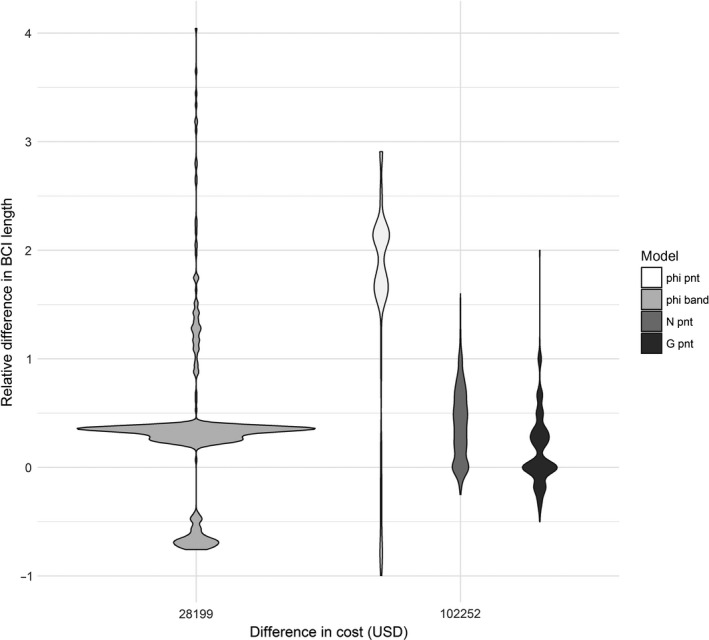
Violin plots showing the difference in relative Bayesian credible interval (BCI) length (a measure of precision) between models built from single and joint data sources using data from a western bluebird case study in ponderosa pine forests within Coconino National Forest in north‐central Arizona, USA between 1999 and 2006 relative to cost (USD) of adding additional data sources. Relative difference in BCI length was calculated as (BCI length single‐BCI length joint)/BCI length joint, so larger numbers equate to more precision gained by incorporating multiple data types. Individual plots show the kernel density distribution across all points sampled. Phi band (left) shows the gain in precision for apparent survival estimates when detection/non‐detection data were added to existing banding data. Phi pnt, N pnt, and G pnt (left to right in right hand group) show the increase in precision of estimates for apparent survival, abundance, and recruitment, respectively, when banding data were added to existing detection/non‐detection data

The largest difference in precision between estimates with single and joint data sources occurred with the apparent survival parameter (mean relative differences in precision for all sampling locations: point compared to joint = 1.615 [*SD* = 0.939], band compared to joint =0.334 [*SD* = 0.714]), with smaller differences observed for abundance (mean relative difference for point compared to joint = 0.394 [*SD* = 0.324]) and recruitment (mean relative difference for point compared to joint = 0.156 [*SD* = 0.318]) (Figure 6). Because apparent survival estimates based on banding data were more precise than apparent survival estimates derived using detection/non‐detection data, there was little gained in precision by adding detection/non‐detection data to banding data (but added cost also was minimal). In contrast, adding banding data to detection/non‐detection data resulted in larger increases in precision, but also required significant increases in cost (Figure 6). Precision also increased for abundance estimates when banding data were added to detection/non‐detection data, but again this addition required a significant cost increase (Figure 6). Adding banding to detection/non‐detection data resulted in minimal changes in precision for recruitment, despite the much higher cost involved (Figure 6).

## 4 DISCUSSION

Demographic models that incorporate multiple data sources are often selected over single source models due to increased precision of resulting population parameter estimates (Besbeas et al., 2002; Schaub & Abadi, 2011). If precision was the only concern for a research program, the increased costs associated with collecting additional data types would not be a consideration. However, most research programs work with limited budgets, and evaluating precision gain versus effort for joint data models is warranted in such studies. This evaluation is particularly important when designing new studies or new phases of projects with multiple possible data collection opportunities. Inherently, some data sources will be more reliable than others, and costs relative to precision vary with the amount of effort required to sample across the landscape. Our general framework to evaluate differences in effort versus precision gain with one type of joint data model can also be applied to joint data models using other combinations of data types in the study design phase.

Our joint model combining occupancy and capture–recapture data yielded parameter estimates that were more precise than those resulting from single data type models, but the increase in precision varied by parameter. The greatest increase in precision estimates occurred for apparent survival probability, which was expected because this parameter was shared by both single data source models. Abundance showed moderate improvement in estimate precision, followed by recruitment.

The amount of precision gained relative to cost also varied by data source. For apparent survival, estimates based on banding data were more precise than those derived from detection/non‐detection data, so little precision was gained (but at minimal cost) when detection/non‐detection data were added to banding data for the joint model. There was a gain, however, in spatial coverage and ability to estimate abundance and recruitment when adding detection/non‐detection data to banding data because banding data were collected at a subset of locations where detection/non‐detection data occurred. Detection/non‐detection data collection is less time‐intensive per area sampled, and a less invasive sampling method, which may be a preferred for sampling threatened and endangered species. Conversely, adding banding to detection/non‐detection data did not increase the spatial scope of inference since banding data were collected at a subset of detection/non‐detection data locations, but resulted in larger increases in precision for survival and abundance. Precision increases with common parameters between data types are expected (Besbeas et al., 2002; Schaub & Abadi, 2011), especially with survival. Costs of adding banding data were high, however. It was possible that our case study had higher costs compared to other types of capture–recapture studies (i.e., small mammal trapping) due to the combination of nest boxes and mist‐nets (although noninvasive DNA capture–recapture methods can be costly). Incorporating demographic data from capture–recapture studies need not be cost‐prohibitive, and are valuable for focused studies (i.e., population viability analyses to inform conservation and management efforts). Therefore, we encourage others to focus on our overall framework to evaluate relative costs to precision increase and assess benefits relative to individual studies and objectives.

One advantage of combining data is that you have a wider range of the covariate space from which to estimate potential effects on demographic parameters. With our study, the covariates selected with the most model support differed between the joint and single data models. The detection/non‐detection data only model had more supported covariates than the joint model, whereas the banding data only model had no supported covariates, suggesting a balance of supported covariates with both data sources for the joint data model. One of our assumptions was that we were sampling randomly from the population for both data sources. Alternatively, data sources may be sampling different portions of the population if there was a discrepancy between supported covariates. In our example, banding data were only collected at nest box locations, whereas detection/non‐detection data were collected at locations with and without nest boxes. The detection/non‐detection model indicated that survival was lower at locations with nest boxes, although this covariate was not supported within the joint data model. Another possibility is that detection/non‐detection and banding data had processes operating at different scales. We limited point‐count detections to 100 m radius, whereas nest box locations (where most banding took place) were located within a 50 m radius of the point‐count station, potentially limiting the number of individuals included within the banding data set, assuming nest boxes did not draw birds in.

A positive relationship with time since fire was evident for initial abundance, apparent survival, and/or recruitment, although results varied between the joint and single data source models. Although uncertainty appeared around what parameter was influenced by time since fire, more certainty existed for the western bluebird population increasing with time since fire. This result is consistent with changes in forest structure occurring after fire and consistent with other studies (Fontaine & Kennedy, 2012; Kotliar et al., 2002; Saab, Russell, & Dudley, 2007).

In the detection/non‐detection only data and joint data models, the snag covariate had a positive relationship for recruitment, but negative with survival. The relationships were not significant, however, with the joint model. Because western bluebirds are cavity‐nesting species, snags are likely to contribute to a positive relationship with recruitment (i.e., Saab, Powell, Kotliar, & Newlon, 2005; Wightman & Germaine, 2006). We did not include interaction effects within the model, but we expected an interaction between snag BA and time since fire because snags decline over time in severely burned areas.

We envision extending the integrated population model to include information on nest data to better inform the recruitment component for a full integrated model. We expect to have increased precision with the recruitment parameter, with no additional costs due to the way these data were collected in this study and parameterized within the cost equation. Depending on data collection limitations, additional data sources will vary in additional costs and effort.

Evaluating value added from different data sources relative to cost will be dependent on the objectives of a given project, as well as available resources. The explicit use of costs within our study establishes a framework for accomplishing this evaluation, and could be coupled with optimization procedures (i.e., Sanderlin, Block, & Ganey, 2014) to maximize accuracy (with a simulation study, i.e., Supporting Information Appendix S1) or precision (like this case study) subject to cost constraints. For example, if estimating recruitment precisely was the primary objective, our case study indicated that adding an additional data source carried high costs but resulted in limited gains in precision. The ability to evaluate costs versus precision, bias, and/or accuracy in parameter estimates is valuable for targeting where to allocate limited resources to meet study objectives and to evaluate power for a given effect size. Further, sampling design trade‐offs not only with or without specific data sources, but different levels of effort with each data source could be evaluated with respect to parameter accuracy and associated costs within our framework, and warrants future exploration. For example, in our simulation study (Supporting Information Appendix S1), both number of banding sites and detection/non‐detection sessions were important for estimating apparent survival (with banding sites being more important), while number of detection/non‐detection sessions was more important for estimating abundance and recruitment. In conclusion, our general framework to evaluate differences in effort versus precision gain with one type of joint data model is applicable to other data type combinations of joint data models, and can also be used to evaluate trade‐offs with different levels of effort within each data source. This framework allows research and monitoring programs to evaluate optimal use of limited funds when multiple data sources are available within the study design phase to meet study objectives.

## CONFLICT OF INTEREST

None declared.

## AUTHORS’ CONTRIBUTIONS

WB and VS conceived the overall project ideas, and JS, WB, and JG conceived the methodological application ideas for the manuscript; JS designed the modeling methods; WB, BS, and VS designed the sampling methodology; WB, BS, and VS collected the data; JS analyzed the data; All authors interpreted the data, JS led the writing of the manuscript. All authors contributed critically to the drafts and gave final approval for publication.

## Data Availability

Data are available through the USDA Forest Service Data Archive (Block, Strohmeyer, & Sanderlin, 2018, https://doi.org/10.2737/RDS-2018-0040). R code for joint and single data models are available as supplementary material (Supporting Information Appendix S4) and for data simulations (Supporting Information Appendix S3).
