# Supplementary material for: Precision gain versus effort with joint models using detection/non‐detection and banding data
Source: Ecol Evol. 2019 Feb 5;9(2):804–17. doi: 10.1002/ece3.4825 (PMC6362443; doi:10.1002/ece3.4825)
Supplement: Supplementary file 1 [file ECE3-9-804-s001.docx]

**Supplemental Information AppendixS1**

**Title**: Precision gain versus effort with joint models using detection/non-detection and banding data

**Author details**: Jamie S. Sanderlin^1,3^, William M. Block^1^, Brenda E. Strohmeyer^1^, Victoria A. Saab^2^, Joseph L. Ganey^1^.

^1^Rocky Mountain Research Station, U.S.D.A. Forest Service, 2500 South Pine Knoll Drive, Flagstaff, Arizona 86001, USA.

^2^Rocky Mountain Research Station, U.S.D.A. Forest Service, Bozeman, Montana 59717, USA.

^3^Corresponding author. email: jlsanderlin@fs.fed.us

**Joint model using detection/non-detection and banding data: assessment of accuracy, bias, percent coverage, and Bayesian credible interval length from a simulation study**

Using a simulation study, we evaluated statistical properties of accuracy, bias, percent coverage, and Bayesian credible interval (BCI) length for abundance, survival, and recruitment estimates using the joint model using detection/non-detection and banding data (described in the main text), with the exception of no covariates to simplify the simulation process. R code for data simulation and JAGS model code can be found in Supplemental Information Text 2.

We based our simulations on our case study of a western bluebird in ponderosa pine forests within Coconino National Forest in north-central Arizona, USA between 1999 and 2006. We assumed the following: constant apparent survival probability (*φ*) for two different levels (0.20 and 0.50), an initial abundance (*λ*) of 5, constant reproduction (*γ*) of 2, constant point count detection probability (*p*_P_) of 0.2, constant banding detection probability of 0.7 (*p*_B_), 5 years of sampling data, 150 number of point count station sampling sites, and an equal probability of banded individuals entering the sample each year. For our simulations, banding locations were not explicitly included in the simulation-estimation process because we did not include location covariates for survival; however, we recognized that number of banding locations is important as a study design component. Therefore, based on our empirical data from the case study, we assumed that 250, 450, and 650 banded individuals equated to approximately 41, 75, and 108 banding sites.

For posterior parameter estimates, we used Bayesian hierarchical models (Gelman, Carlin, Stern, & Rubin, 2004) in JAGS (Plummer, 2003) using the *rjags* package in R (R Core Team, 2017). We also used package *jagsUI* (Kellner, 2016) a wrapper around *rjags* to implement parallel processing, and package *coda* (Plummer, Best, Cowles, & Vines, 2006) to obtain posterior parameter estimates. We used independent non-informative priors: *λ* ~ Uniform (0,10), *γ* ~ Uniform (0, 10), *φ* ~ Uniform (0, 1), *p*_P_  ~ Uniform (0, 1) and *p*_B_  ~ Uniform (0, 1). We ran 3 parallel chains (total length of 50,000 *iterations* [*it*], burn-in 20,000 *it*, thinning 10 *it*) to estimate the posterior distribution median of model parameters and 95% Bayesian Credible Intervals (BCI) for each replicate of the simulation Markov chain Monte Carlo (MCMC) process. Convergence was reached with all replicates of simulations (<1.1 [Brooks & Gelman, 1998]). We used 200 replicates for each simulation combination to evaluate frequentist statistical properties of relative root mean square error (RRMSE), relative bias (RBIAS), percent coverage, and BCI length for apparent survival probability, abundance, and recruitment.

We calculated the average RRMSE, RBIAS, and BCI length for all location by year combinations of parameter estimates, since we were interested in overall metrics. We used RRMSE as a measure of accuracy (equation 1), and defined RRMSE as:

$RRMSE= \frac{\sqrt{\left( \frac{1}{r} \right)\sum_{l=1}^{r} \left( \hat{\theta}_{l}-\theta_{l} \right)^{2}}}{\bar{\theta}},$ eqn 1

where *r* was the number of replicates, $\hat{\theta}_{l}$was the estimated parameter posterior median at replicate *l*, $\theta_{l}$was the true parameter at replicate *l*, and $\bar{\theta}$ was the mean of the true parameter values over all replicates. Using the same notation, we defined RBIAS (equation 2) as:

$RBIAS= \frac{\left( \left( \frac{1}{r} \right)\sum_{l=1}^{r} \left( \hat{\theta}_{l}-\theta_{l} \right) \right)}{\bar{\theta}}.$ eqn 2

Percent coverage was calculated as the number of times the true parameter value was contained within the 95% BCI for each replicate out of the total number of replicates.

We examined study design parameters for both detection/non-detection and banding data sources (2 levels [3, 5] of number of sampling sessions for point counts, 3 levels [250, 450, 650] of number of banded individuals which equates to ~ 30, 75, 120 banding sites) and biological uncertainty (different levels of constant apparent survival probability – low [0.20], mid [0.50]), for a total of 12 different simulation combination levels. The parameter space included approximate levels from our 8-year study (e.g., 3 sampling sessions for point counts, 450 banded individuals, and 0.50 constant survival probability).

Percent coverage was high (near the nominal 95% frequentist level) for recruitment and apparent survival for all design scenarios (Supp. Fig. 1.1), but abundance was lower (~0.85), with higher percent coverage with fewer sessions. BCI length decreased with increased effort from both point count sessions and number of banding sites for apparent survival (Supp. Fig. 1.2). There was increased precision (decreased RRMSE) with higher levels of true apparent survival. Precision increased more with additional banding sites compared to more sessions for collecting detection/non-detection data. BCI length decreased with increased effort from point count sessions, but not number of banding sites, for recruitment and abundance (Supp. Figs. 1.3, 1.4). There was increased precision when true apparent survival was lower for recruitment and abundance. Relative bias decreased for abundance and recruitment with increased effort with point count sessions, but not number of banding sites (Supp. Fig. 1.5). Relative bias was close to zero for true apparent survival was 0.5, and slightly positive when apparent survival was 0.2 (there was also decreased relative bias with increased effort from number of banding sites). Both abundance and recruitment were negatively biased.

Accuracy increased with increased effort from number of banding sites and slightly from number of point count sessions for apparent survival (Supp. Fig. 1.6). Relative root mean square error was higher for true apparent survival of 0.2 than 0.5. Accuracy increased with number of point count sessions, but not number of banding sites, for abundance (Supp. Fig. 1.7). However, accuracy decreased with increased number of point count sessions for recruitment (Supp. Fig. 1.8). There was increased accuracy for true apparent survival of 0.5 compared to 0.2 for both abundance and recruitment (Supp. Figs. 1.7). Results from our simulation study indicated that inference from our joint model was valid for percent coverage, precision, relative bias and accuracy, since our parameter space was contained within these simulation scenarios. Our simulation results also indicate some interesting trade-offs with sampling design for different data sources and warrants future exploration.

**References**

Brooks, S. P., & Gelman, A. (1998). General methods for monitoring convergence of iterative simulations. *Journal of Computational and Graphical Statistics*, *7*(4), 434–455. doi:10.1080/10618600.1998.10474787

Gelman, A., Carlin, J. B., Stern, H. S., & Rubin, D. B. (2004). *Bayesian data analysis* (2nd ed.). New York: Chapman and Hall/CRC.

Kellner, K. (2016). jagsU: a wrapper around “jags” to streamline ‘JAGS’ analyses. *JR Package Version 1.4.2. Https://CRAN.R-Project.Org/Package=jagsUI’, JagsUI: A Wrapper Around ‘Rjags’ to Streamline ‘JAGS’ Analyses. R Package Version 1.4.2. Https://CRAN.R-Project.Org/Package=jagsUI.* Retrieved from https://cran.r-project.org/web/ packages/jagsUI/index.html

Plummer, M. (2003). JAGS: A program for analysis of Bayesian graphical models using Gibbs sampling. *Proceedings of the 3rd International Workshop on Distributed Statistical Computing (DSC 2003)*, 20–22. doi:10.1.1.13.3406

Plummer, M., Best, N., Cowles, K., & Vines, K. (2006). CODA: Convergence diagnosis and output analysis for MCMC. *R News*, *6*(1), 7–11. doi:http: // CRAN. R-project. org/ package= coda .

R Core Team. (2017). R: A language and environment for statistical computing. Vienna, Austria: R Foundation for Statistical Computing. Retrieved from https://www.r-project.org


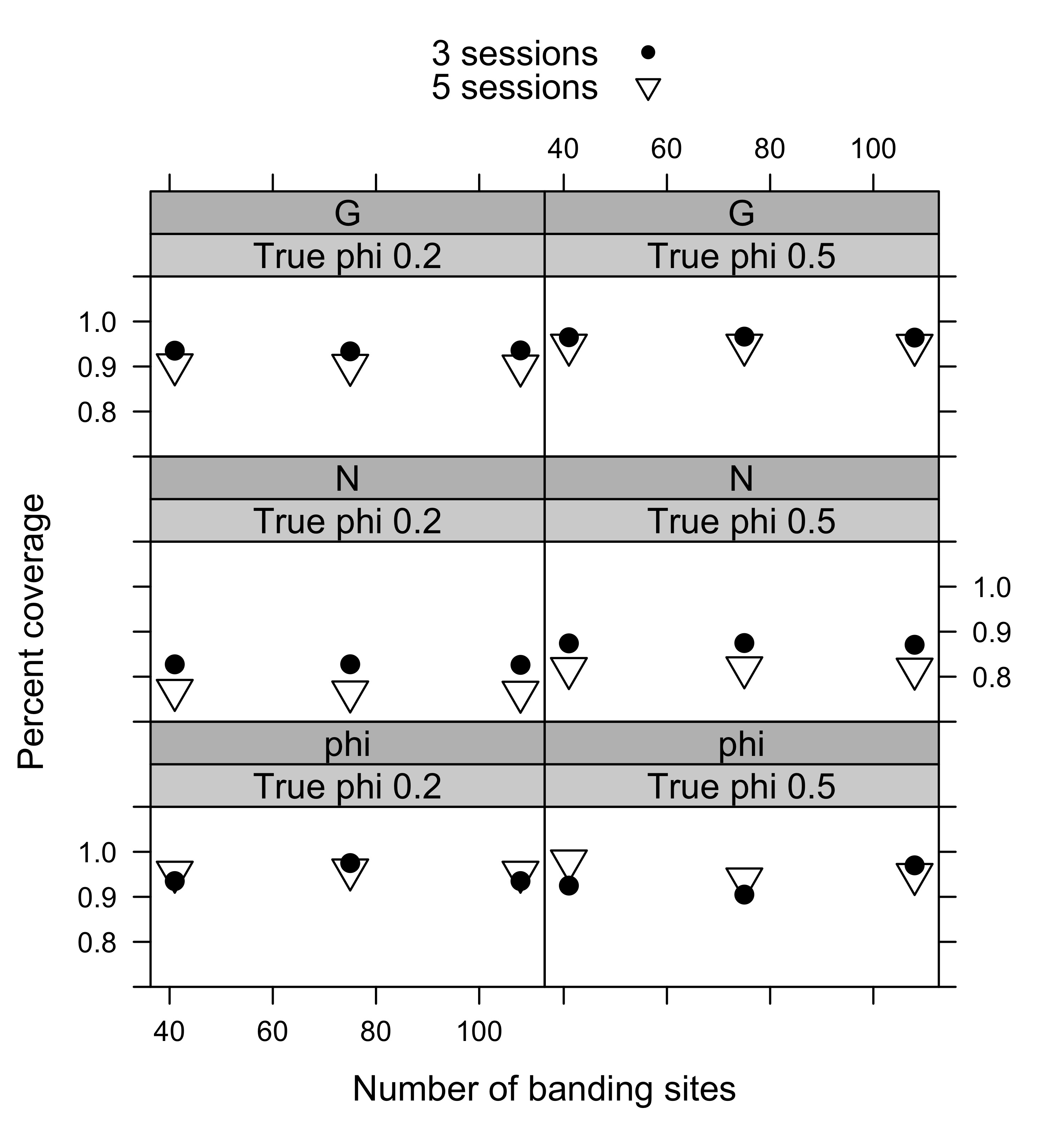


Supplementary Fig 1.1. Percent coverage of apparent survival (*φ*), abundance (N), and recruitment (*G*) from a simulation study based on a western bluebird case study in ponderosa pine forests within Coconino National Forest in north-central Arizona, USA between 1999 and 2006. Design scenarios included 2 levels of point count sampling sessions, and 3 levels of banding sites. True parameters for apparent survival had 2 levels.


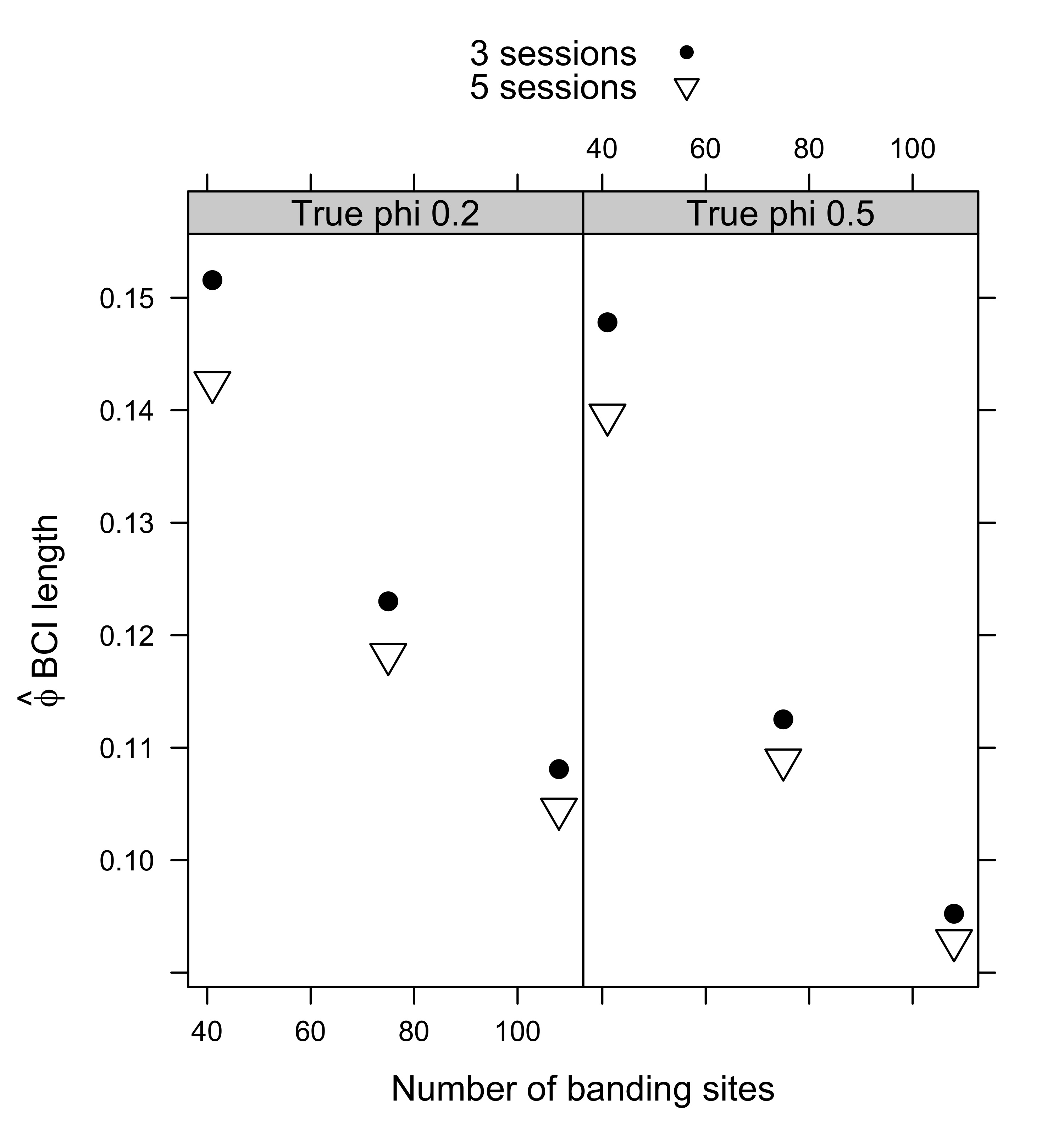
Supplementary Fig 1.2. Bayesian credible interval (BCI) length of apparent survival (*φ*) from a simulation study based on a western bluebird case study in ponderosa pine forests within Coconino National Forest in north-central Arizona, USA between 1999 and 2006. Design scenarios included 2 levels of point count sampling sessions, and 3 levels of banding sites. True parameters for apparent survival had 2 levels.


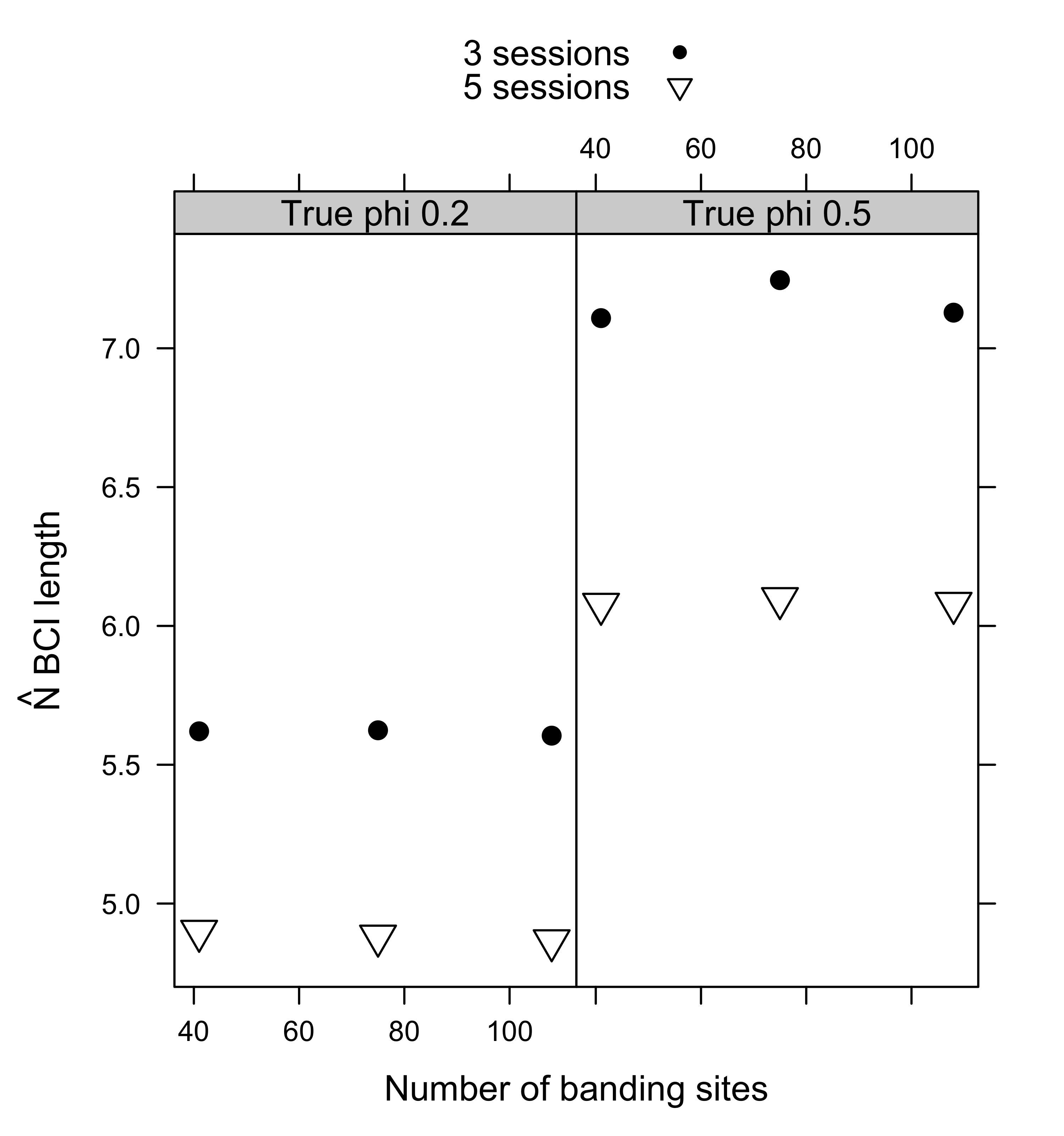


Supplementary Fig 1.3. Bayesian credible interval (BCI) length of abundance (*N*) from a simulation study based on a western bluebird case study in ponderosa pine forests within Coconino National Forest in north-central Arizona, USA between 1999 and 2006. Design scenarios included 2 levels of point count sampling sessions, and 3 levels of banding sites. True parameters for apparent survival had 2 levels.


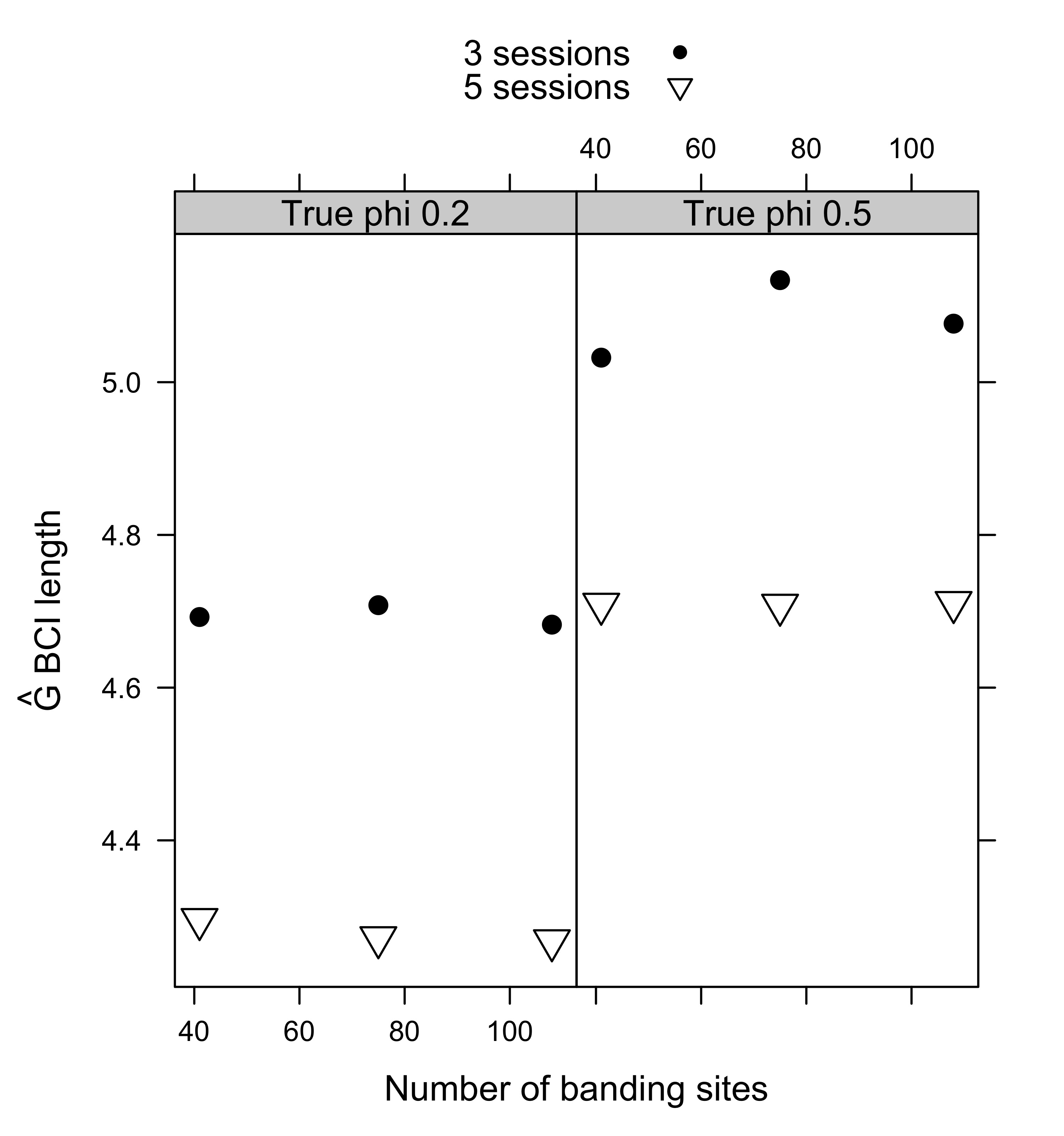


Supplementary Fig 1.4. Bayesian credible interval (BCI) length of recruitment (*G*) from a simulation study based on a western bluebird case study in ponderosa pine forests within Coconino National Forest in north-central Arizona, USA between 1999 and 2006. Design scenarios included 2 levels of point count sampling sessions, and 3 levels of banding sites. True parameters for apparent survival had 2 levels.


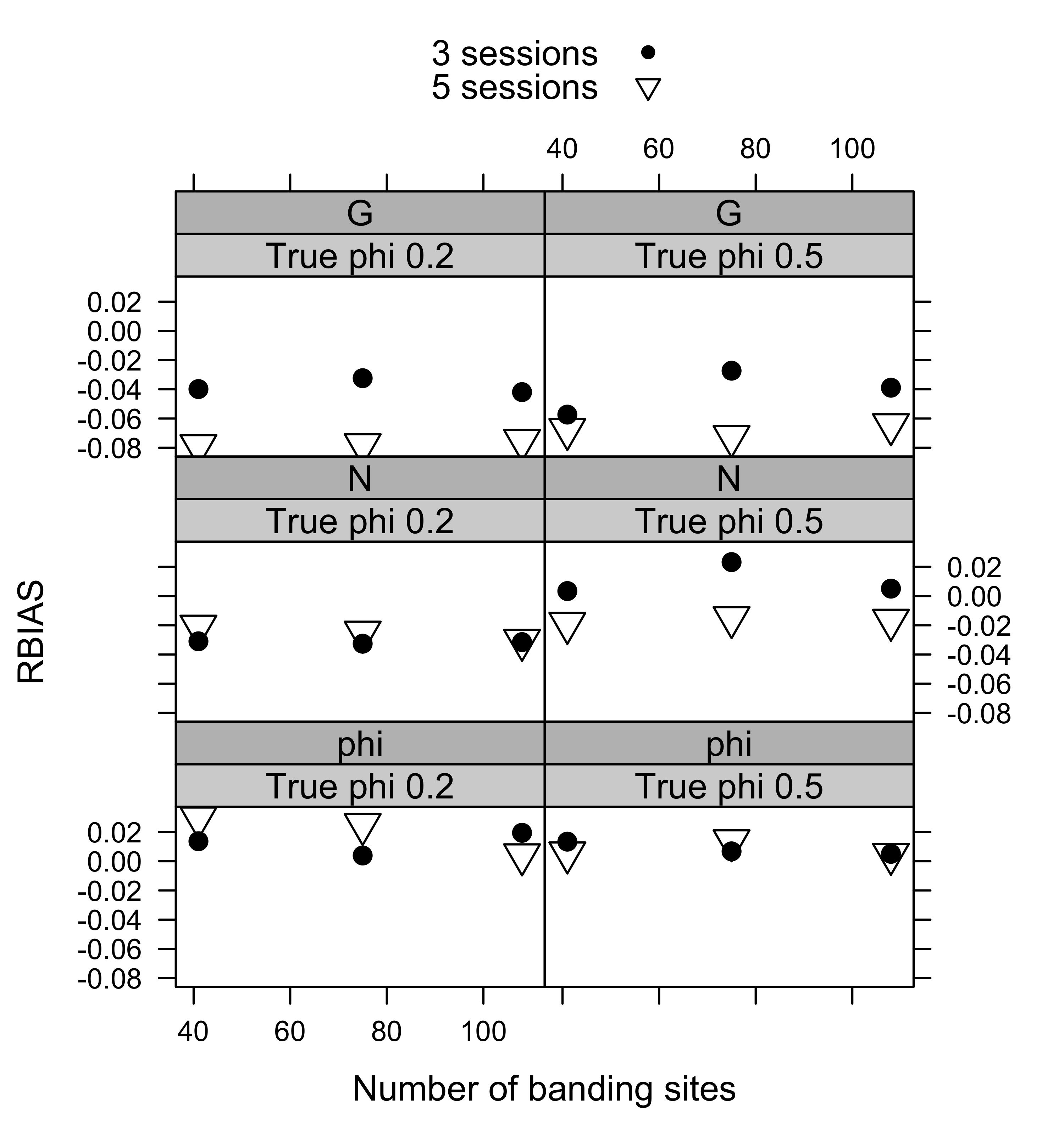
Supplementary Fig 1.5. Relative bias (RBIAS) of apparent survival (*φ*), abundance (N), and recruitment (*G*) from a simulation study based on a western bluebird case study in ponderosa pine forests within Coconino National Forest in north-central Arizona, USA between 1999 and 2006. Design scenarios included 2 levels of point count sampling sessions, and 3 levels of banding sites. True parameters for apparent survival had 2 levels.


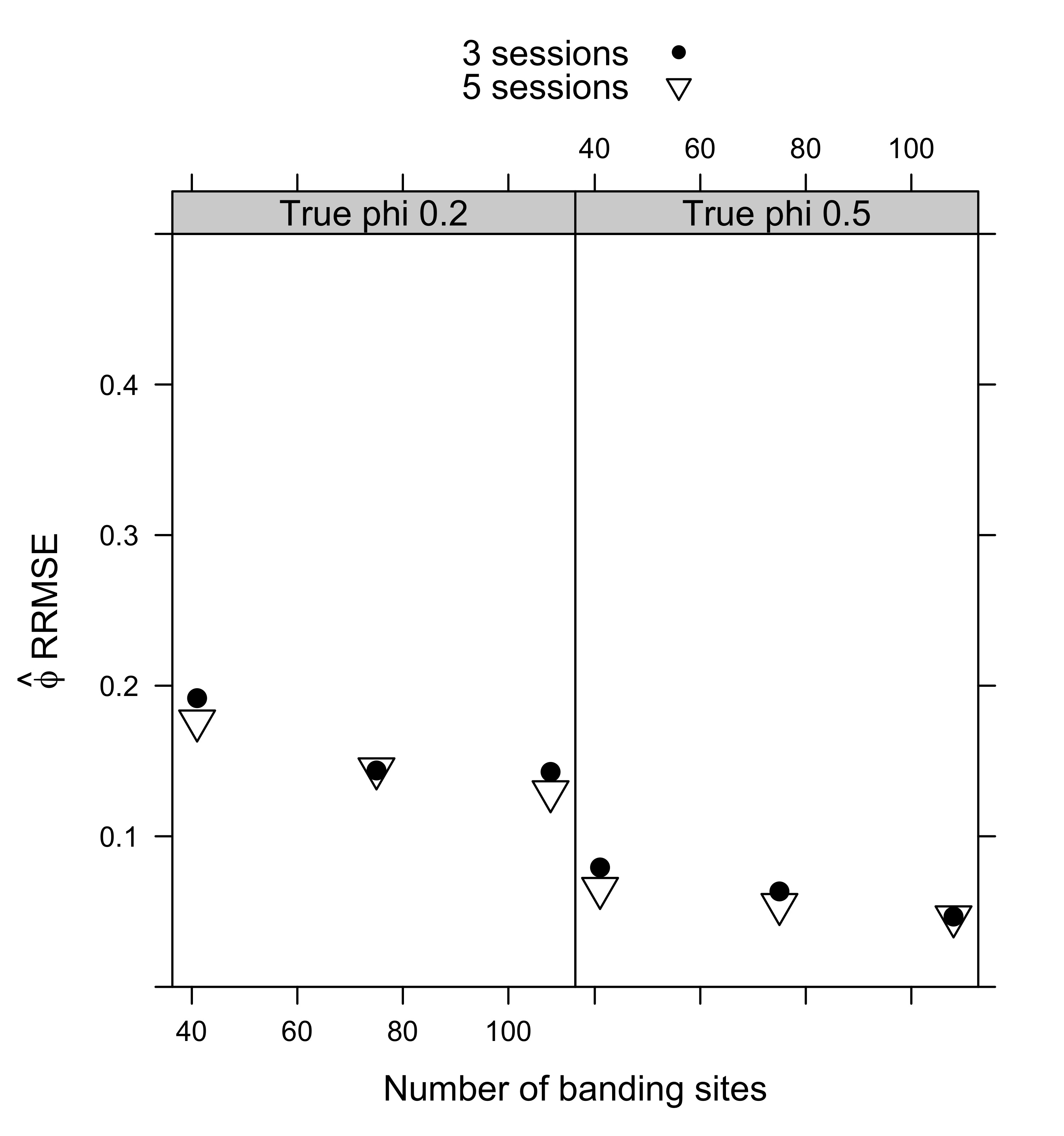


Supplementary Fig 1.6. Relative root mean square error (RRMSE) of apparent survival (*φ*) from a simulation study based on a western bluebird case study in ponderosa pine forests within Coconino National Forest in north-central Arizona, USA between 1999 and 2006. Design scenarios included 2 levels of point count sampling sessions, and 3 levels of banding sites. True parameters for apparent survival had 2 levels.


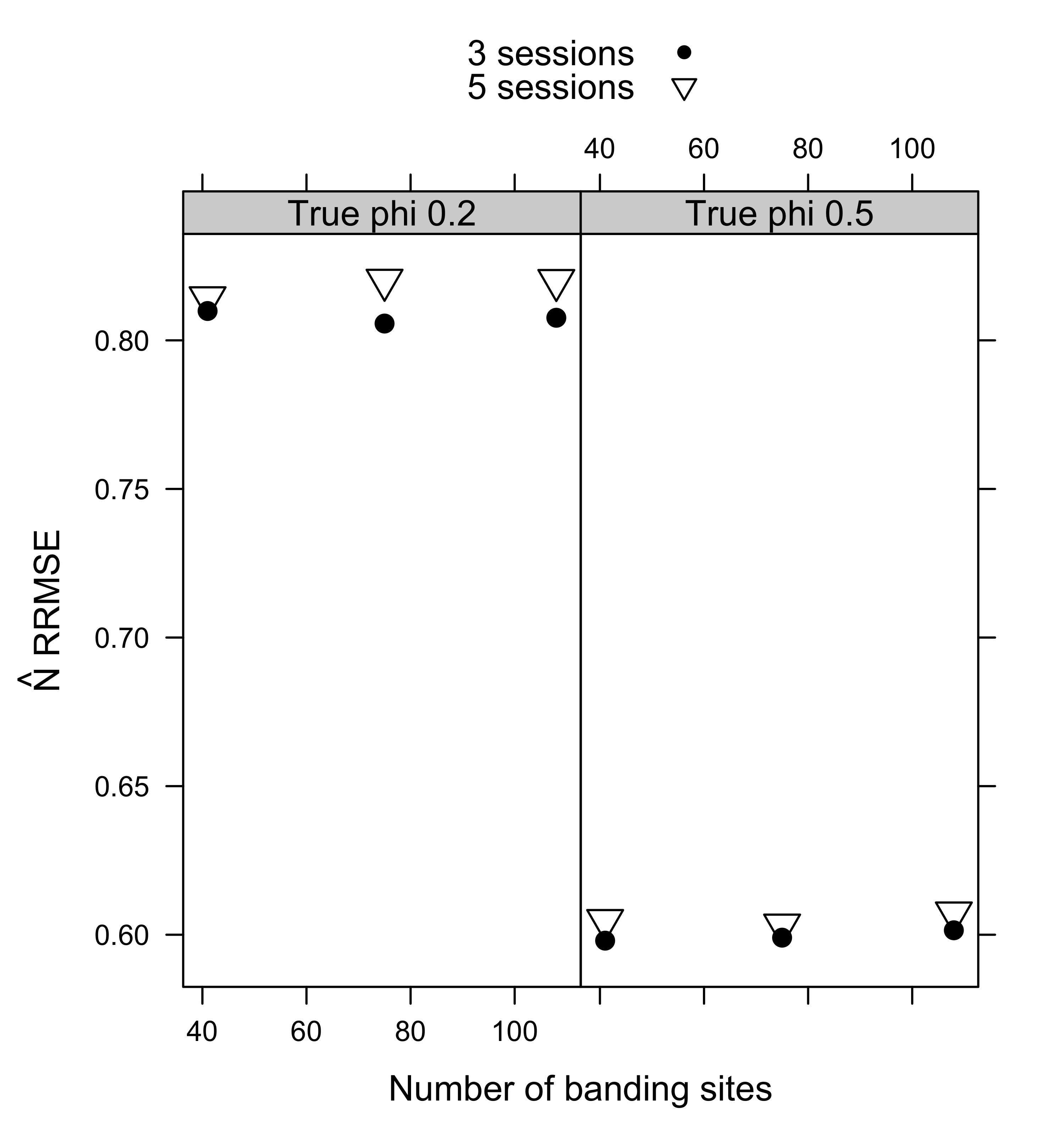


Supplementary Fig 1.7. Relative root mean square error (RRMSE) of abundance (*N*) from a simulation study based on a western bluebird case study in ponderosa pine forests within Coconino National Forest in north-central Arizona, USA between 1999 and 2006. Design scenarios included 2 levels of point count sampling sessions, and 3 levels of banding sites. True parameters for apparent survival had 2 levels.


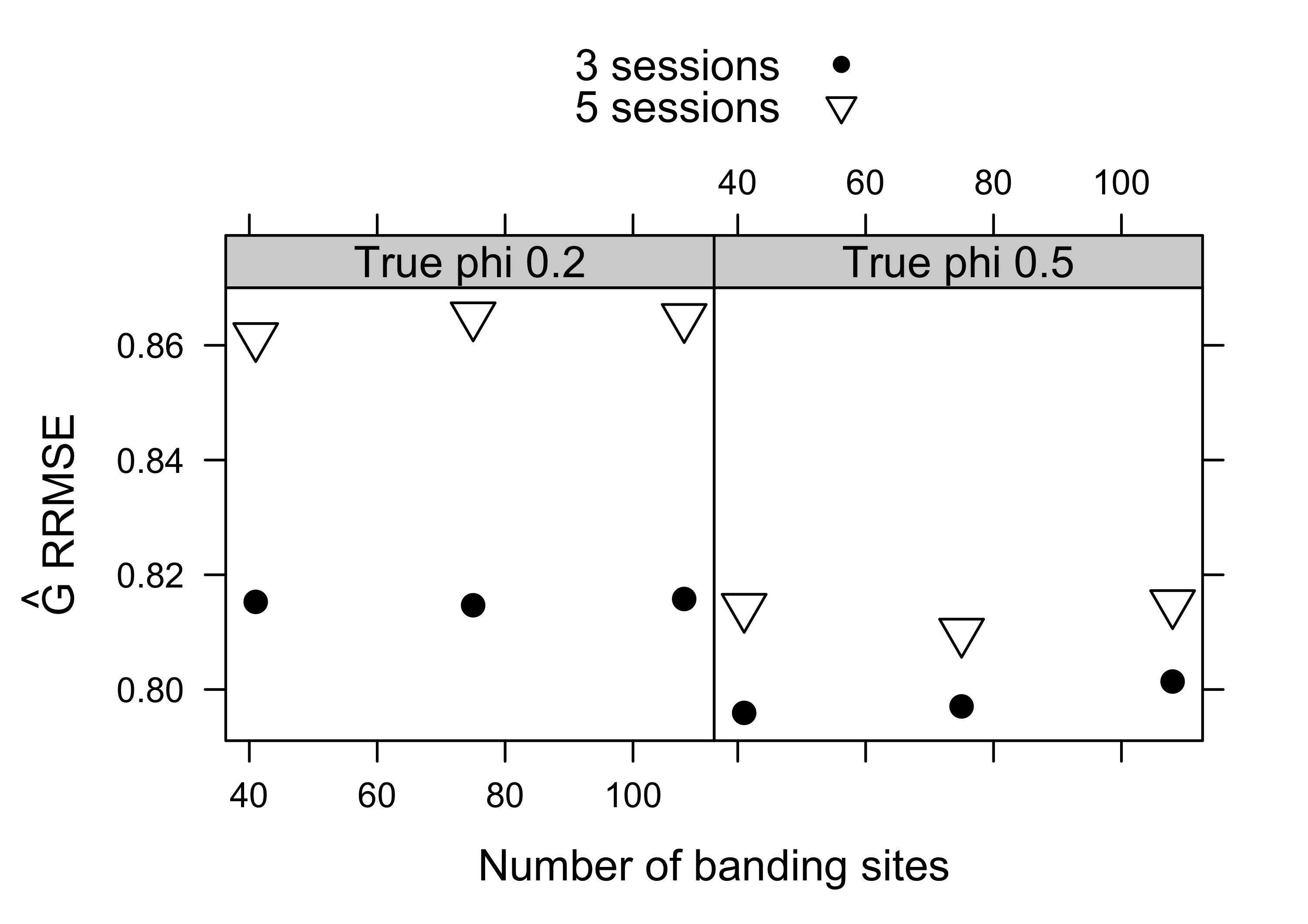


Supplementary Fig 1.8. Relative root mean square error (RRMSE) of recruitment (*G*) from a simulation study based on a western bluebird case study in ponderosa pine forests within Coconino National Forest in north-central Arizona, USA between 1999 and 2006. Design scenarios included 2 levels of point count sampling sessions, and 3 levels of banding sites. True parameters for apparent survival had 2 levels.
