# Supplementary material for: Precision gain versus effort with joint models using detection/non‐detection and banding data
Source: Ecol Evol. 2019 Feb 5;9(2):804–17. doi: 10.1002/ece3.4825 (PMC6362443; doi:10.1002/ece3.4825)
Supplement: Supplementary file 2 [file ECE3-9-804-s002.docx]

**Supplemental Information AppendixS2**

**Title**: Precision gain versus effort with joint models using detection/non-detection and banding data

**Author details**: Jamie S. Sanderlin^1,3^, William M. Block^1^, Brenda E. Strohmeyer^1^, Victoria A. Saab^2^, Joseph L. Ganey^1^.

^1^Rocky Mountain Research Station, U.S.D.A. Forest Service, 2500 South Pine Knoll Drive, Flagstaff, Arizona 86001, USA.

^2^Rocky Mountain Research Station, U.S.D.A. Forest Service, Bozeman, Montana 59717, USA.

^3^Corresponding author. email: jlsanderlin@fs.fed.us

**R code for joint model simulations (data simulations)**

#Simulation for banding and point count data

#------------------------------------------------------------

#data simulation parameters loosely based on Western Bluebird

#study in northern Arizona, USA

#------------------------------------------------------------

sim <- function(){

#-----Model Parameters---------------------------------------

phi <- 0.5 #constant survival

lambda <- 5 #initial abundance per location

gam <- 2 #constant reproduction

p.p <- 0.2 #constant point count detection probability (per site, session, year)

p.b <- 0.7 #constant banding detection probability

nyears <- 5 #number of years

nsites <- 150 #number of locations (cells,pnts,etc.)

first.p <- 1/nyears #equal probability of banded individuals entering sample each year

p.first <- rep(first.p,nyears)

nsess <- 3 #number of point count sessions (3 or 5)

nind <- 450 #number of banded individuals (250 or 450 or 650)

#note: banding locations not explicitly included in simulation/estimation

#because we are not including location covariates for survival, but

#number of locations for banding is important in the study design

#component, thus we assume that 250 individuals correspond to ~30 sites,

#450 individuals correspond to ~75 sites, and 650 individuals correspond

#to ~120 sites.

#------------------------------------------------------------

cat.rand <- function(cp=cp) {

cat.vector <- rmultinom(1,1,cp)

out <- which(cat.vector==1)

out

}

#-----Additional parameters------------

N.true <- array(NA,dim=c(nyears,nsites))

p.site.true <- array(NA,dim=c(nyears,nsites,nsess))

S.true <- array(NA,dim=c(nyears-1,nsites))

G.true <- array(NA,dim=c(nyears-1,nsites))

Z.true <- array(NA,dim=c(nind,nyears))

first <- rep(NA,nind)

#--------------------------------------

#data matrices

Y.pnt <- array(NA,dim=c(nyears,nsites,nsess))

Y.band <- array(0,dim=c(nind,nyears))

for (k in 1:nsites) {

#time 1

N.true[1,k] <- rpois(1,lambda) #initial abundance

#-----point count detection-----

for (j in 1:nsess){

p.site.true[1,k,j] <- 1-((1-p.p)^N.true[1,k])

Y.pnt[1,k,j] <- rbinom(1,1,p.site.true[1,k,j])

}

#time > 1

for (t in 2:nyears){

S.true[t-1,k] <- rbinom(1,N.true[t-1,k],phi) #number survived

G.true[t-1,k] <- rpois(1,gam) #number recruited

N.true[t,k] <- S.true[t-1,k] + G.true[t-1,k]

#-----point count detection-----

for (j in 1:nsess){

p.site.true[t,k,j] <- 1-((1-p.p)^N.true[t,k])

Y.pnt[t,k,j] <- rbinom(1,1,p.site.true[t,k,j])

}

}

}

#--------banding data-------------

for(i in 1:nind){

#simulate when individuals enter sample

first[i] <- cat.rand(cp=p.first)

#individuals enter sample with probability 1

Z.true[i,first[i]] <- 1

Y.band[i,first[i]] <- 1

if (first[i]<nyears){

for (t in (first[i]+1):nyears){

mu1 <- phi*Z.true[i,t-1]

Z.true[i,t] <- rbinom(1,1,mu1)

mu2 <- p.b*Z.true[i,t]

Y.band[i,t] <- rbinom(1,1,mu2)

}

}

}

list(nsites=nsites, nyears=nyears,nsess=nsess,nind=nind,

Y.pnt=Y.pnt, Y.band=Y.band,first=first,N.true=N.true,

S.true=S.true, G.true=G.true)

}

**R code for joint model simulations (JAGS model)**

#dynamic occupancy model + CJS survival model

model {

#priors

lambda ~ dunif(0,10) #initial abundance

p.p ~ dunif(0,1) #point count detection

phi ~ dunif(0,1) #survival probability

gam ~ dunif(0,10) #reproduction

p.b ~ dunif(0,1) #banding detection

for (k in 1:nsites) {

#time 1

N[1,k]~dpois(lambda) #abundance

#-----point count detection-----

for (j in 1:nsess){

p.site[1,k,j] <- 1-pow((1-p.p),N[1,k])

Y.pnt[1,k,j] ~ dbern(p.site[1,k,j])

}

#time > 1

for (t in 2:nyears){

S[t-1,k] ~ dbin(phi,N[t-1,k]) #number that survived

G[t-1,k] ~ dpois(gam) #number that were recruited

N[t,k]<- S[t-1,k] + G[t-1,k]

#-----point count detection-----

for (j in 1:nsess){

p.site[t,k,j] <- 1-pow((1-p.p),N[t,k])

Y.pnt[t,k,j] ~ dbern(p.site[t,k,j])

}

}

}

#--------banding data-------------

for(i in 1:nind){

#individuals enter sample with probability 1

Z[i,first[i]] ~ dbern(1)

for (t in (first[i]+1):nyears){

mu1[i,t] <- phi*Z[i,t-1]

Z[i,t] ~ dbern(mu1[i,t])

mu2[i,t] <- p.b*Z[i,t]

Y.band[i,t] ~ dbern(mu2[i,t])

}

}

}
