# Supplementary material for: Precision gain versus effort with joint models using detection/non‐detection and banding data
Source: Ecol Evol. 2019 Feb 5;9(2):804–17. doi: 10.1002/ece3.4825 (PMC6362443; doi:10.1002/ece3.4825)
Supplement: Supplementary file 3 [file ECE3-9-804-s003.docx]

**Supplemental Information AppendixS3**

**Title**: Precision gain versus effort with joint models using detection/non-detection and banding data

**Author details**: Jamie S. Sanderlin^1,3^, William M. Block^1^, Brenda E. Strohmeyer^1^, Victoria A. Saab^2^, Joseph L. Ganey^1^.

^1^Rocky Mountain Research Station, U.S.D.A. Forest Service, 2500 South Pine Knoll Drive, Flagstaff, Arizona 86001, USA.

^2^Rocky Mountain Research Station, U.S.D.A. Forest Service, Bozeman, Montana 59717, USA.

^3^Corresponding author. email: jlsanderlin@fs.fed.us

**Joint model using detection/non-detection and banding data: assessment of independence assumption**

One of the critical components for factoring the component likelihoods of the joint likelihood (equation 12, main text) was the assumption of independence among data types. This assumption is often difficult to meet when small populations have different demographic data structures that contain the same individuals (Schaub & Abadi, 2011), and may overestimate precision (Anderson et al., 1994). However, the more independent datasets are, there is a greater chance of not having individuals with the same demography (Schaub & Abadi, 2011), another critical assumption for combining data sources with common parameters.

It is possible that the same individuals were present in both detection/non-detection and banding data sets, so we assessed differences in posterior medians and Bayesian credible interval (BCI) length of the joint model (described in the main text) with and without using data from point count stations that had nest boxes. We assessed these differences for apparent survival (*φ*), abundance (*N*), and recruitment (*G*). For the joint model using point count data that was not collected at nest boxes, we used posterior predictive distributions to obtain parameter estimates for locations that had nest boxes for *φ*, *N*, and *G*. A simulation study (i.e., Abadi et al. [2010]) would also assist with identifying potential impacts from violation of the independence assumption on parameter estimates from the joint model.

Median posterior estimates for apparent survival from the joint model were similar with and without data from point count stations that had nest boxes (Sup. Fig. 3.1), with median differences ranging from -0.03 to 0.05. There were more locations that had larger posterior medians from the joint model using data from point count stations that had nest boxes than without. In contrast, median posterior estimates for abundance showed more differences (Sup. Fig. 3.2) from the joint model using data with and without point count stations that had nest boxes. Most locations had larger posterior medians from the joint model without using data from point count stations that had nest boxes. Similar to abundance, recruitment also showed more differences than apparent survival, but fewer differences than abundance, from the joint model using data with and without point count stations that had nest boxes (Sup. Fig. 3.3). Again, there were more locations that had larger posterior medians from the joint model without data from point count stations that had nest boxes, although there were several locations that had identical posterior medians.

Lengths of 95% BCIs for apparent survival from the joint model using data from point count stations that had nest boxes were longer than with the joint model without data from point count stations that had nest boxes (Sup. Fig. 3.4), indicating that apparent survival estimates from the joint model using data without point count stations that had nest boxes had more precision. There were several locations with very similar BCI lengths (on the line). In contrast, most points had lower precision for abundance at locations from the joint model without using data from point count stations that had nest boxes (Sup. Fig. 3.5), and some locations even had really wide BCIs (i.e., >25). Finally, there was also less precision for recruitment at locations from the joint model using data from point count stations that had nest boxes (Sup. Fig. 3.6), and some locations with really wide BCIs (i.e., >20).

While using data from point count stations without nest boxes reduces (or even eliminates) dependency among data sources, it would reduce data available by half in our case study, severely limiting the ability to make inference at large spatial and temporal scales. Rossman et al (2016) indicated that at least 75 survey sites and 5 years of data are needed for reliable estimates using dynamic N-occupancy models, although we saw very large reductions in precision for abundance and recruitment with 77 survey sites and 8 years of data using the joint model without point count data from locations that had nest boxes. With fewer data, posterior medians for abundance and recruitment were a lot higher than with survival (potentially indicating differences in bias), likely because survival was also being informed by the other data source (banding data). However, apparent survival estimates using data without point count stations that had nest boxes had more precision, which could be due to differences between survival estimates from banding versus detection/non-detection data in our case study. With our case study, lack of independence (if any) did not seem to increase survival precision, but instead appeared to decrease precision when all data were used within the joint model. Precision appeared to increase with abundance and recruitment when all data were used, but this may be more a function of the increase in spatial replicates than lack of independence.

**References**

Abadi, F., Gimenez, O., Arlettaz, R. & Schaub, M. 2010. An assessment of integrated population models: bias, accuracy, and violation of the assumption of independence. *Ecology* 91:7-14.

Anderson, D.R., Burnhm, K.P., & White, G.C. 1994. AIC model selection in overdispersed capture-recapture data. *Ecology* 75:1780-1793.

Rossman, S., Yackulic, C. B., Saunders, S. P., Reid, J., Davis, R., & Zipkin, E. F. (2016). Dynamic N-occupancy models: estimating demographic rates and local abundance from detection-nondetection data. *Ecology*, *97*(12), 3300–3307.

Schaub, M., & Abadi, F. (2011). Integrated population models: a novel analysis framework for deeper insights into population dynamics. *Journal of Ornithology*, *152*(Suppl 1), S227–S237. doi:10.1007/s10336-010-0632-7.


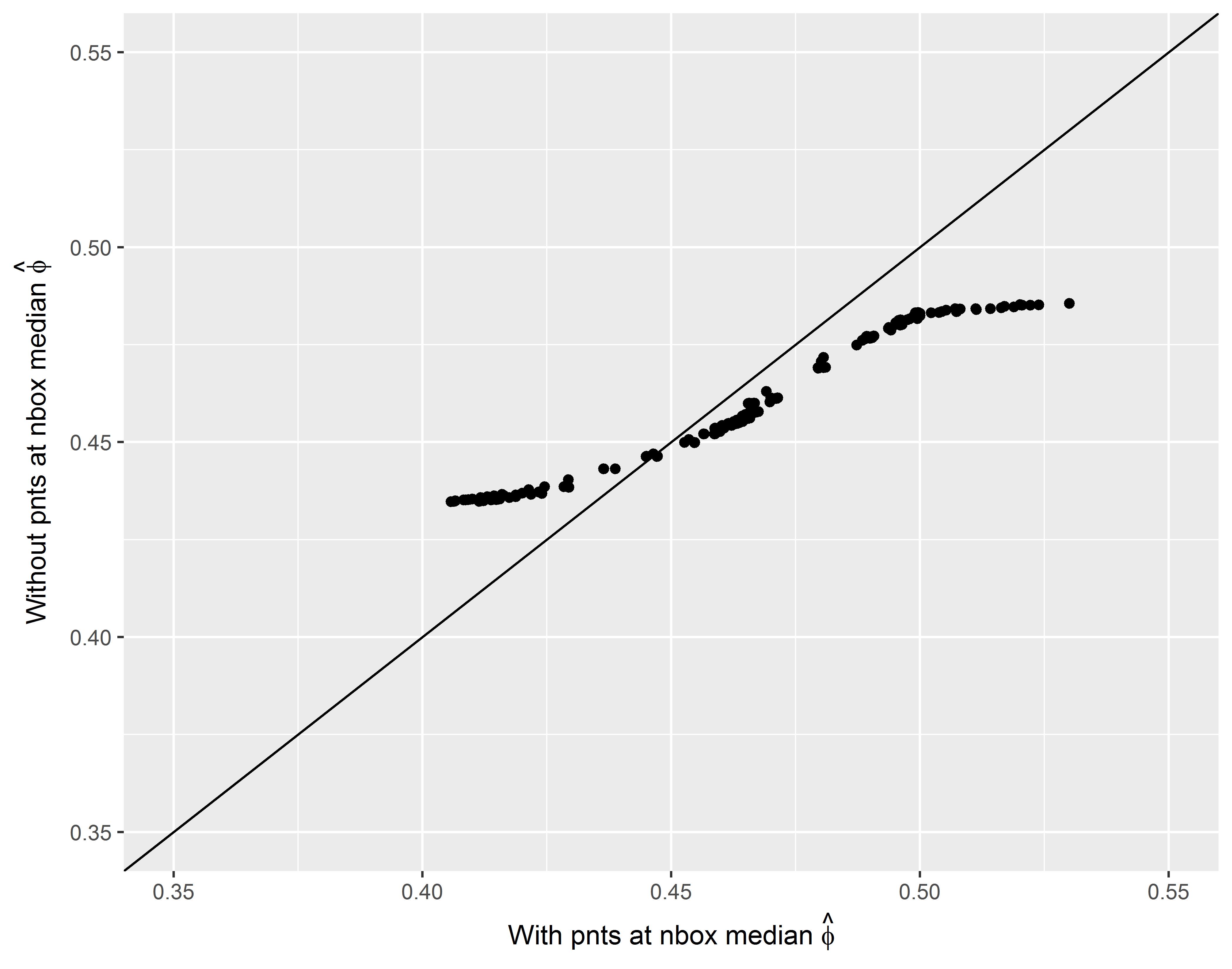


Supplementary Fig 3.1. Comparison of posterior medians of apparent survival (*φ*) using point count data collected at all sampling locations (*with pnts at nbox*) and point count data collected only at locations without nest boxes (*without pnts at nbox*) of a western bluebird case study in ponderosa pine forests within Coconino National Forest in north-central Arizona, USA between 1999 and 2006. The line indicates a ‘1 to 1’ relationship between medians, where points below the line indicate medians from point count data that had nest boxes were larger than medians from point count data that did not have nest boxes, and vice versa for points above the line.


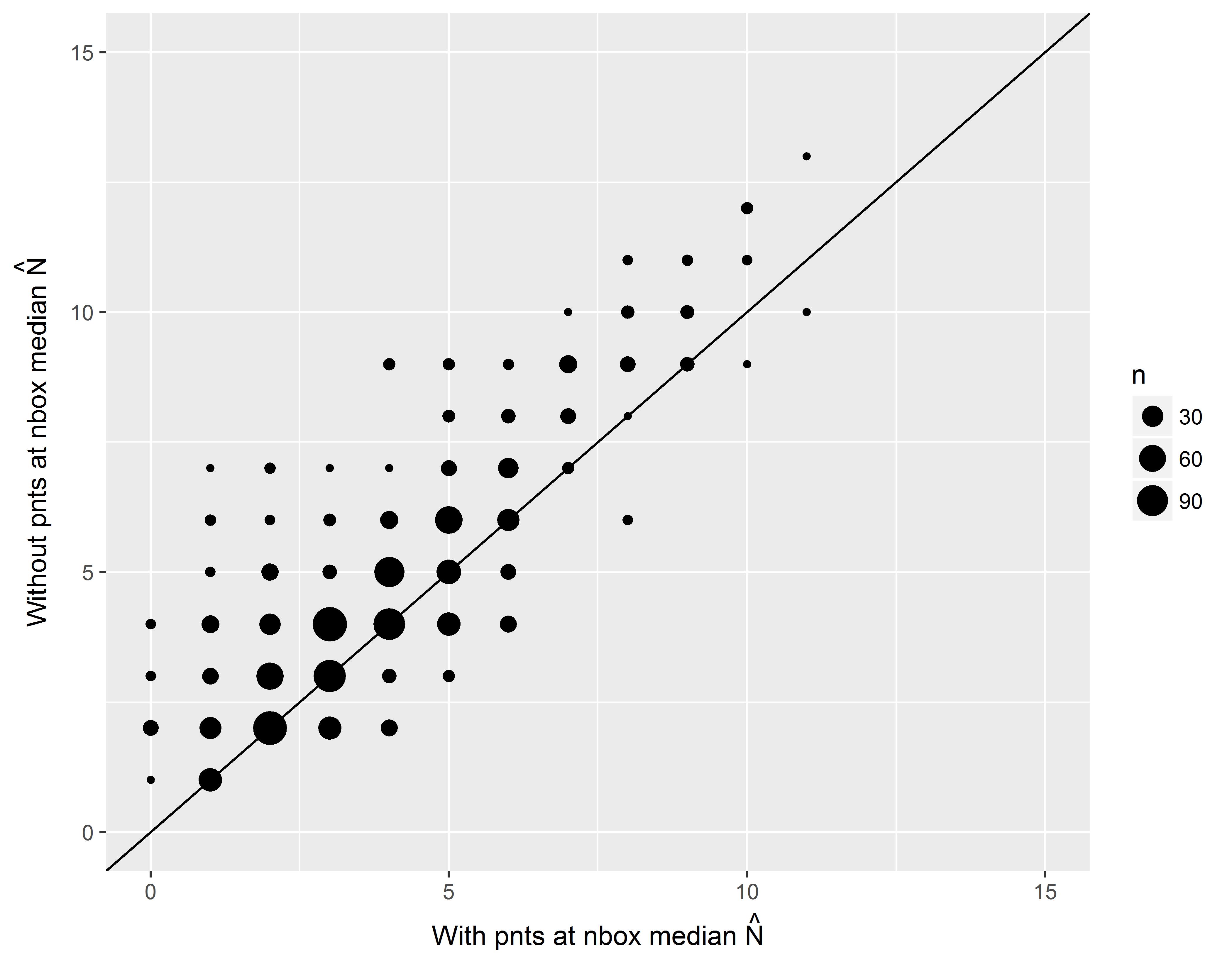


Supplementary Fig 3.2. Comparison of posterior medians of abundance (*N*) using point count data collected at all sampling locations (*with pnts at nbox*) and point count data collected only at locations without nest boxes (*without pnts at nbox*) of a western bluebird case study in ponderosa pine forests within Coconino National Forest in north-central Arizona, USA between 1999 and 2006. Dot size indicates the count (*n*) of locations with associated *x* and *y* coordinates. The line indicates a ‘1 to 1’ relationship between medians, where points below the line indicate medians from point count data that had nest boxes were larger than medians from point count data that did not have nest boxes, and vice versa for points above the line.


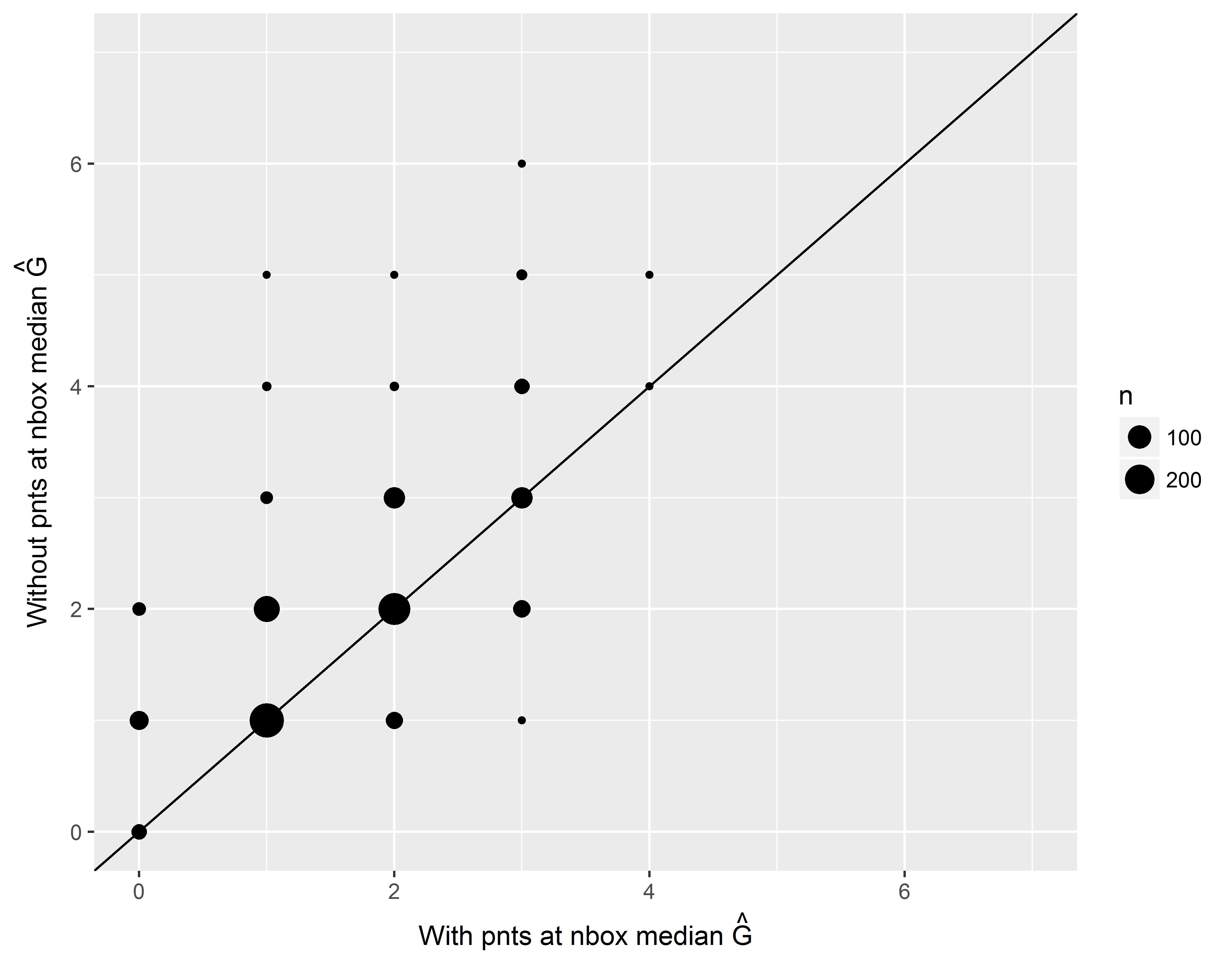


Supplementary Fig 3.3. Comparison of posterior medians of recruitment (*G*) using point count data collected at all sampling locations (*with pnts at nbox*) and point count data collected only at locations without nest boxes (*without pnts at nbox*) of a western bluebird case study in ponderosa pine forests within Coconino National Forest in north-central Arizona, USA between 1999 and 2006. Dot size indicates the count (*n*) of locations with associated *x* and *y* coordinates. The line indicates a ‘1 to 1’ relationship between medians, where points below the line indicate medians from point count data that had nest boxes were larger than medians from point count data that did not have nest boxes, and vice versa for points above the line.


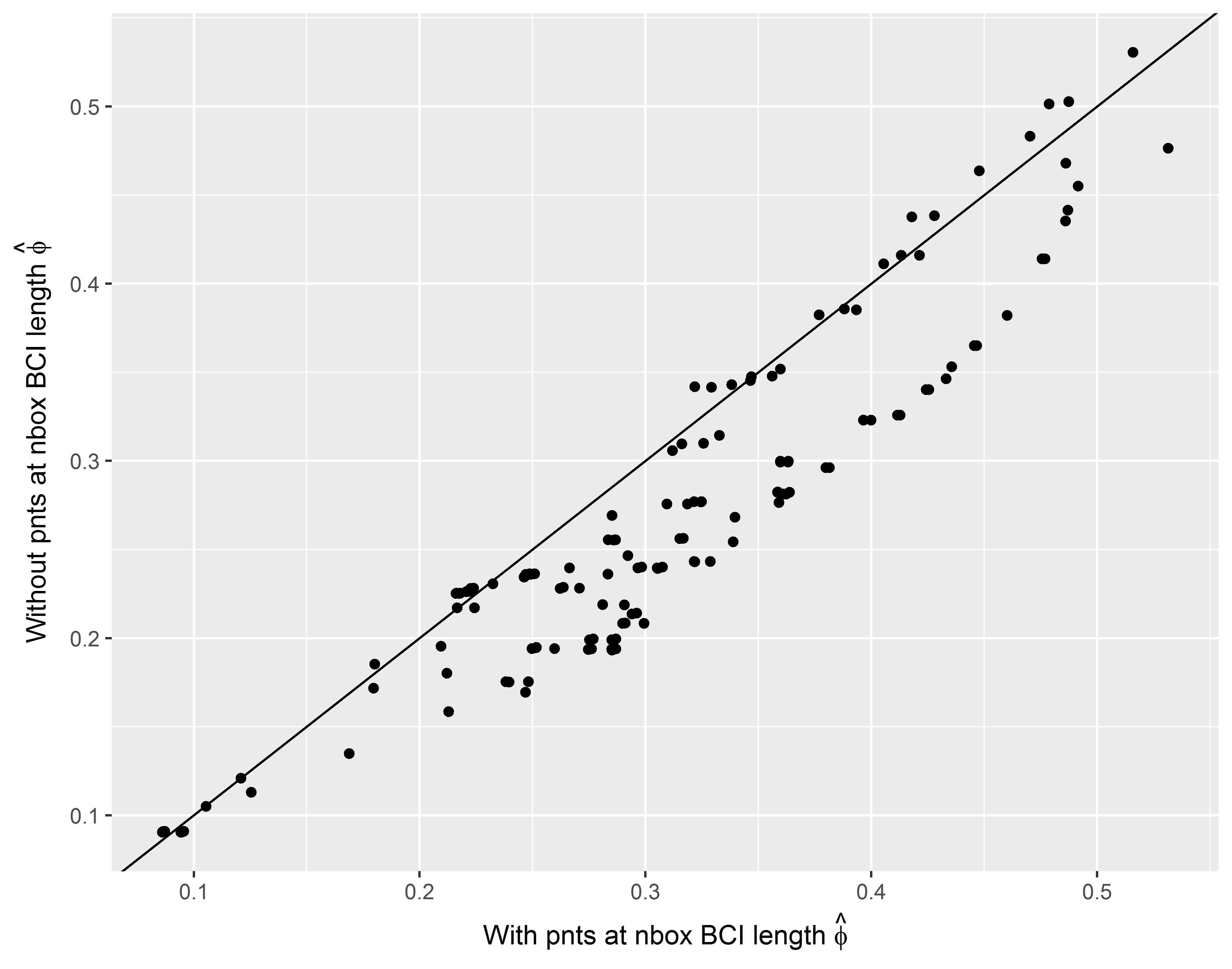


Supplementary Fig 3.4. Comparison of Bayesian credible interval (BCI) length of apparent survival (*φ*) using point count data collected at all sampling locations (*with pnts at nbox*) and point count data collected only at locations without nest boxes (*without pnts at nbox*) of a western bluebird case study in ponderosa pine forests within Coconino National Forest in north-central Arizona, USA between 1999 and 2006. The line indicates a ‘1 to 1’ relationship between BCI lengths, where points below the line indicate lower precision from point count data that had nest boxes than from point count data that did not have nest boxes, and vice versa for points above the line.


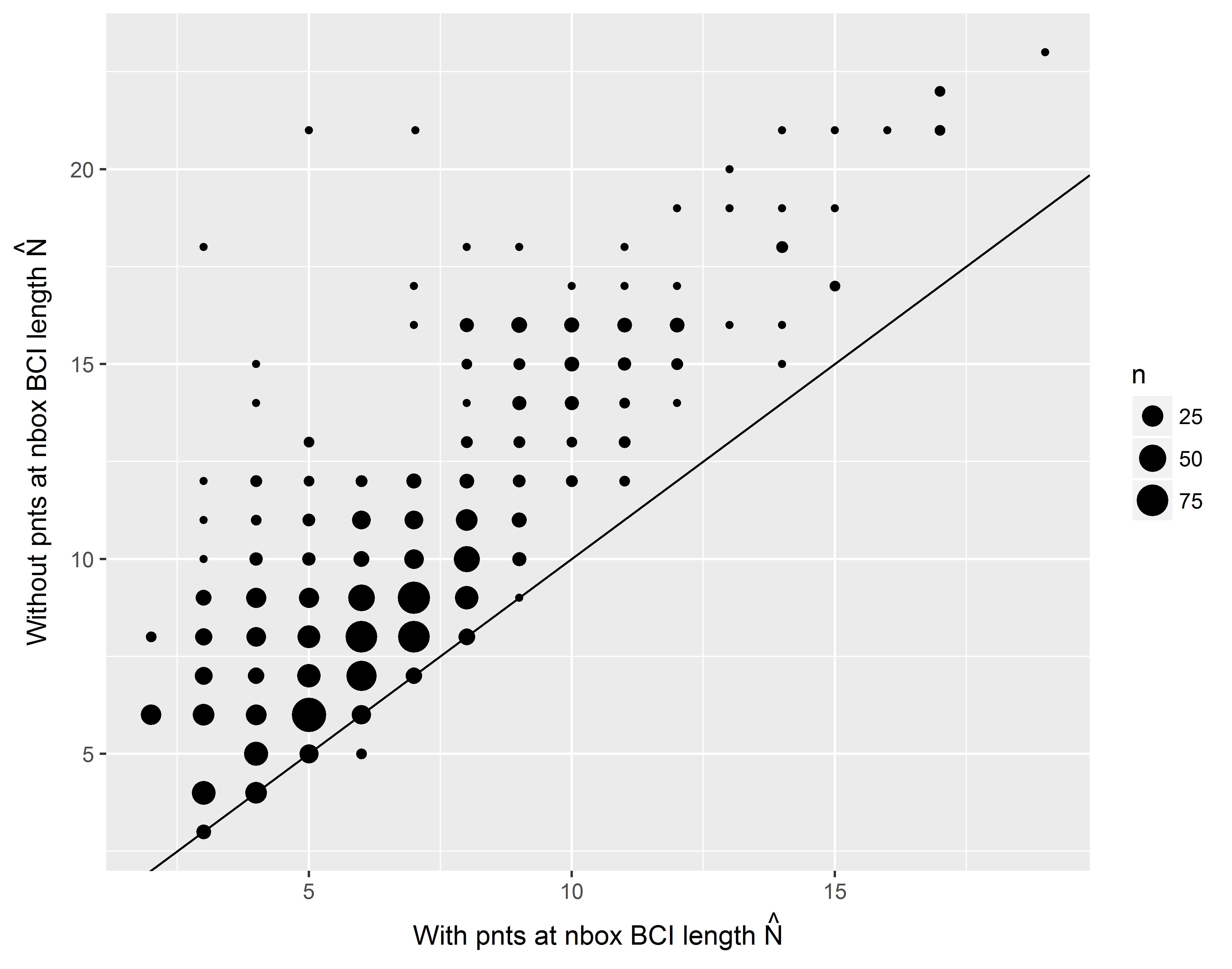


Supplementary Fig 3.5. Comparison of Bayesian credible interval (BCI) length of abundance (*N*) using point count data collected at all sampling locations (*with pnts at nbox*) and point count data collected only at locations without nest boxes (*without pnts at nbox*) of a western bluebird case study in ponderosa pine forests within Coconino National Forest in north-central Arizona, USA between 1999 and 2006. Dot size indicates the count (*n*) of locations with associated *x* and *y* coordinates. The line indicates a ‘1 to 1’ relationship between BCI lengths, where points below the line indicate lower precision from point count data that had nest boxes than from point count data that did not have nest boxes, and vice versa for points above the line.


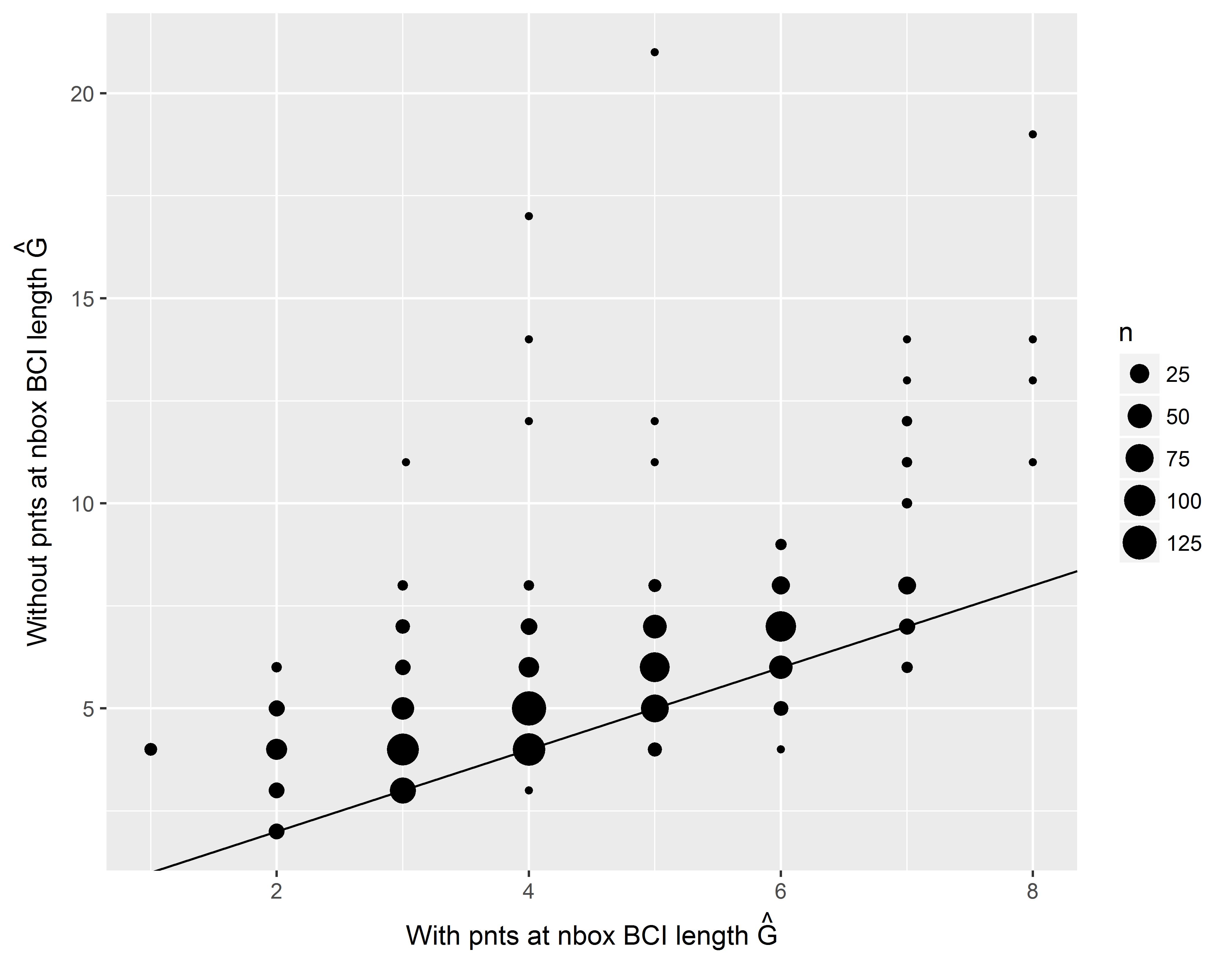


Supplementary Fig 3.6. Comparison of Bayesian credible interval (BCI) length of recruitment (*G*) using point count data collected at all sampling locations (*with pnts at nbox*) and point count data collected only at locations without nest boxes (*without pnts at nbox*) of a western bluebird case study in ponderosa pine forests within Coconino National Forest in north-central Arizona, USA between 1999 and 2006. Dot size indicates the count (*n*) of locations with associated *x* and *y* coordinates. The line indicates a ‘1 to 1’ relationship between BCI lengths, where points below the line indicate lower precision from point count data that had nest boxes than from point count data that did not have nest boxes, and vice versa for points above the line.
