# Supplementary material for: Precision gain versus effort with joint models using detection/non‐detection and banding data
Source: Ecol Evol. 2019 Feb 5;9(2):804–17. doi: 10.1002/ece3.4825 (PMC6362443; doi:10.1002/ece3.4825)
Supplement: Supplementary file 4 [file ECE3-9-804-s004.docx]

**Supplemental Information AppendixS4**

**Title**: Precision gain versus effort with joint models using detection/non-detection and banding data

**Author details**: Jamie S. Sanderlin^1,3^, William M. Block^1^, Brenda E. Strohmeyer^1^, Victoria A. Saab^2^, Joseph L. Ganey^1^.

^1^Rocky Mountain Research Station, U.S.D.A. Forest Service, 2500 South Pine Knoll Drive, Flagstaff, Arizona 86001, USA.

^2^Rocky Mountain Research Station, U.S.D.A. Forest Service, Bozeman, Montana 59717, USA.

^3^Corresponding author. email: jlsanderlin@fs.fed.us

**R code for point-count data only dynamic-N occupancy model**

model {

#priors

a0 ~ dnorm(0,0.1)

a1latent[2] ~ dnorm(0,0.1)

a1latent[1] ~ dnorm(0.0003,0.000001)

a2latent[2] ~ dnorm(0,0.1)

a2latent[1] ~ dnorm(-0.006,0.000001)

a3latent[2] ~ dnorm(0,0.1)

a3latent[1] ~ dnorm(0.08,0.000001)

sigma.a4 ~ dunif(0,5)

tau.a4 <- pow(sigma.a4,-2)

#random effect of transect

for (i in 1:15){

a4latent[i,2] ~ dnorm(0,tau.a4)

a4latent[i,1] ~ dnorm(0,0.3)

a4[i] <-a4latent[i,za4+1]

alpha4[i] <-za4*a4[i]

}

b0 ~ dunif(0,1)

lb0 <- log(b0)/(1-log(b0))

b1latent[2] ~ dnorm(0,0.1)

b1latent[1] ~ dnorm(1.1,0.000001)

sigma.b2 ~ dunif(0,5)

tau.b2 <- pow(sigma.b2,-2)

#random effect of observer

for (i in 1:9){

b2latent[i,2] ~ dnorm(0,tau.b2)

b2latent[i,1] ~ dnorm(-2.2,0.1)

b2[i] <- b2latent[i,zb2+1]

beta2[i] <- zb2*b2[i]

}

c0 ~ dunif(0,1)

lc0 <- log(c0)/(1-log(c0))

c1latent[2] ~ dnorm(0,0.1)

c1latent[1] ~ dnorm(-0.67,0.000001)

c2latent[2] ~ dnorm(0,0.1)

c2latent[1] ~ dnorm(0.2,0.000001)

c3latent[2] ~ dnorm(0,0.1)

c3latent[1] ~ dnorm(0.3,0.000001)

c4latent[2] ~ dnorm(0,0.1)

c4latent[1] ~ dnorm(-2.0,0.000001)

d0 ~ dnorm(0,0.1)

d1latent[2] ~ dnorm(0,0.1)

d1latent[1] ~ dnorm(0.006,0.000001)

d2latent[2] ~ dnorm(0,0.1)

d2latent[1] ~ dnorm(0.004,0.000001)

d3latent[2] ~ dnorm(0,0.1)

d3latent[1] ~ dnorm(0.02,0.000001)

d4latent[2] ~ dnorm(0,0.1)

d4latent[1] ~ dnorm(0.32,0.000001)

#indicator variable selection

za1 ~ dbern(0.5)

za2 ~ dbern(0.5)

za3 ~ dbern(0.5)

za4 ~ dbern(0.5)

zb1 ~ dbern(0.5)

zb2 ~ dbern(0.5)

zc1 ~ dbern(0.5)

zc2 ~ dbern(0.5)

zc3 ~ dbern(0.5)

zc4 ~ dbern(0.5)

zd1 ~ dbern(0.5)

zd2 ~ dbern(0.5)

zd3 ~ dbern(0.5)

zd4 ~ dbern(0.5)

a1 <-a1latent[za1+1]

a2 <-a2latent[za2+1]

a3 <-a3latent[za3+1]

b1 <-b1latent[zb1+1]

c1 <-c1latent[zc1+1]

c2 <-c2latent[zc2+1]

c3 <-c3latent[zc3+1]

c4 <-c4latent[zc4+1]

d1 <-d1latent[zd1+1]

d2 <-d2latent[zd2+1]

d3 <-d3latent[zd3+1]

d4 <-d4latent[zd4+1]

alpha1 <-za1*a1

alpha2 <-za2*a2

alpha3 <-za3*a3

beta1 <-zb1*b1

chi1 <-zc1*c1

chi2 <-zc2*c2

chi3 <-zc3*c3

chi4 <-zc4*c4

delta1 <-zd1*d1

delta2 <-zd2*d2

delta3 <-zd3*d3

delta4 <-zd4*d4

for (k in 1:nsites) {

#time 1

log(lambda[k]) <- a0+alpha1*dnbr[k]+alpha2*nbox[k]+alpha3*tfire[k,1]+alpha4[transect[k]]

N[1,k]~dpois(lambda[k]) #abundance

for (j in 1:nsess){

logit(p[1,k,j]) <- lb0 + beta1*nbox[k] + beta2[obs[1,k,j]] #detection

temp[1,k,j] <- 1-pow((1-p[1,k,j]),N[1,k])

Y[1,k,j] ~ dbern(temp[1,k,j])

#_____Bayesian GOF_________

exp_y[1,k,j] <- temp[1,k,j]

#simulated data under model

ynew[1,k,j] ~ dbern(temp[1,k,j])

#squared loss

loss_data[1,k,j] <- pow((exp_y[1,k,j]-Y[1,k,j]),2)

loss_sim[1,k,j] <- pow((exp_y[1,k,j]-ynew[1,k,j]),2)

}

#time > 1

for (t in 2:nyears){

logit(phi[t-1,k]) <- lc0 + chi1*nbox[k] + chi2*tfire[k,t] + chi3*live[t,k] + chi4*snag[t,k] #survival

S[t-1,k] ~ dbin(phi[t-1,k],N[t-1,k]) #number that survived

log(gam[t-1,k]) <- d0 + delta1*nbox[k] + delta2*tfire[k,t] + delta3*live[t,k] + delta4*snag[t,k] #reproduction

G[t-1,k] ~ dpois(gam[t-1,k])

N[t,k]<- S[t-1,k] + G[t-1,k]

for (j in 1:nsess){

logit(p[t,k,j]) <- b0 + beta1*nbox[k] + beta2[obs[t,k,j]] #detection

temp[t,k,j] <- 1-pow((1-p[t,k,j]),N[t,k])

Y[t,k,j] ~ dbern(temp[t,k,j])

#_____Bayesian GOF_________

exp_y[t,k,j] <- temp[t,k,j]

#simulated data under model

ynew[t,k,j] ~ dbern(temp[t,k,j])

#squared loss

loss_data[t,k,j] <- pow((exp_y[t,k,j]-Y[t,k,j]),2)

loss_sim[t,k,j] <- pow((exp_y[t,k,j]-ynew[t,k,j]),2)

}

}

}

#Bayesian GOF

sum_data_loss <-sum(loss_data[,,])

sum_sim_loss <-sum(loss_sim[,,])

#test statistics should be ~0.5 if model fits

test<-step(sum_sim_loss-sum_data_loss)

}

**R code for banding data only Cormack-Jolly Seber model**

model {

#priors

#mean capture probability (p)

p0~dunif(0,1)

lp0<- log(p0/(1-p0)) #logit transformation

a2latent[2] ~ dnorm(0,0.1) #live tree term for survival probability

a2latent[1] ~ dnorm(-0.006,0.000001)

a3latent[2] ~ dnorm(0,0.1) #snag term for survival probability

a3latent[1] ~ dnorm(0.08,0.000001)

a4latent[2] ~ dnorm(0,0.1) #time since fire term for survival probability

a4latent[1] ~ dnorm(0.08,0.000001)

b1latent[2] ~ dnorm(0,0.1) #sex term for capture probability

b1latent[1] ~ dnorm(1.1,0.000001)

#mean survival probability (phi)

phi0 ~ dunif(0,1)

lphi0 <- log(phi0/(1-phi0)) #logit transformation

for(t in 1:(nyears-1)){

#survival

for (k in 1:nlocs){

logit(phi[t,k])<-lphi0 + alpha2*live[t,k] + alpha3*snag[t,k]

+ alpha4*tfire[k,t]

}

}

#indicator variable selection

za2 ~ dbern(0.5)

za3 ~ dbern(0.5)

za4 ~ dbern(0.5)

zb1 ~ dbern(0.5)

a2 <-a2latent[za2+1]

a3 <-a3latent[za3+1]

a4 <-a4latent[za4+1]

b1 <-b1latent[zb1+1]

alpha2 <-za2*a2

alpha3 <-za3*a3

alpha4 <-za4*a4

beta1 <-zb1*b1

#######################################################

for(i in 1:nind){

#detection

logit(p[i])<-lp0 + beta1*sex[i]

#individuals enter sample with probability 1

Z[i,first[i]] ~ dbern(1)

for (t in 1:first[i]){

loss.data[i,t]<-0

loss.sim[i,t]<-0

}

for (t in (first[i]+1):nyears){

mu1[i,t] <- phi[t-1,loc[i]]*Z[i,t-1]

Z[i,t] ~ dbern(mu1[i,t])

mu2[i,t] <- p[i]*Z[i,t]

Y[i,t] ~ dbern(mu2[i,t])

#_____Bayesian GOF_________

exp_y[i,t] <- mu2[i,t]

#simulated data under model

ynew[i,t] ~ dbern(mu2[i,t])

#squared loss

loss.data[i,t] <- pow((exp_y[i,t]-Y[i,t]),2)

loss.sim[i,t] <- pow((exp_y[i,t]-ynew[i,t]),2)

}

}

#Bayesian GOF

sum.data.loss <-sum(loss.data[,])

sum.sim.loss <-sum(loss.sim[,])

#test statistics should be ~0.5 if model fits

test<-step(sum.sim.loss-sum.data.loss)

}

**R code for joint point-count and banding data model**

model {

#priors

a0 ~ dnorm(0,0.1)

a1latent[2] ~ dnorm(0,0.1)

a1latent[1] ~ dnorm(0.0003,0.000001)

a2latent[2] ~ dnorm(0,0.1)

a2latent[1] ~ dnorm(-0.006,0.000001)

a3latent[2] ~ dnorm(0,0.1)

a3latent[1] ~ dnorm(0.08,0.000001)

sigma.a4 ~ dunif(0,5)

tau.a4 <- pow(sigma.a4,-2)

#random effect of transect

for (i in 1:15){

a4latent[i,2] ~ dnorm(0,tau.a4)

a4latent[i,1] ~ dnorm(0,0.3)

a4[i] <-a4latent[i,za4+1]

alpha4[i] <-za4*a4[i]

}

b0 ~ dunif(0,1)

lb0 <- log(b0)/(1-log(b0))

b1latent[2] ~ dnorm(0,0.1)

b1latent[1] ~ dnorm(1.1,0.000001)

sigma.b2 ~ dunif(0,5)

tau.b2 <- pow(sigma.b2,-2)

#random effect of observer for point count detection

for (i in 1:9){

b2latent[i,2] ~ dnorm(0,tau.b2)

b2latent[i,1] ~ dnorm(-2.2,0.1)

b2[i] <- b2latent[i,zb2+1]

beta2[i] <- zb2*b2[i]

}

#mean detection probability for banding

pb0~dunif(0,1)

lpb0<- log(pb0/(1-pb0)) #logit transformation

c0 ~ dunif(0,1)

lc0 <- log(c0)/(1-log(c0))

c1latent[2] ~ dnorm(0,0.1)

c1latent[1] ~ dnorm(-0.67,0.000001)

c2latent[2] ~ dnorm(0,0.1)

c2latent[1] ~ dnorm(0.2,0.000001)

c3latent[2] ~ dnorm(0,0.1)

c3latent[1] ~ dnorm(0.3,0.000001)

c4latent[2] ~ dnorm(0,0.1)

c4latent[1] ~ dnorm(-2.0,0.000001)

d0 ~ dnorm(0,0.1)

d1latent[2] ~ dnorm(0,0.1)

d1latent[1] ~ dnorm(0.006,0.000001)

d2latent[2] ~ dnorm(0,0.1)

d2latent[1] ~ dnorm(0.004,0.000001)

d3latent[2] ~ dnorm(0,0.1)

d3latent[1] ~ dnorm(0.02,0.000001)

d4latent[2] ~ dnorm(0,0.1)

d4latent[1] ~ dnorm(0.32,0.000001)

e1latent[2] ~ dnorm(0,0.1) #sex term for capture probability

e1latent[1] ~ dnorm(0.53,0.000001)

#indicator variable selection

za1 ~ dbern(0.5)

za2 ~ dbern(0.5)

za3 ~ dbern(0.5)

za4 ~ dbern(0.5)

zb1 ~ dbern(0.5)

zb2 ~ dbern(0.5)

zc1 ~ dbern(0.5)

zc2 ~ dbern(0.5)

zc3 ~ dbern(0.5)

zc4 ~ dbern(0.5)

zd1 ~ dbern(0.5)

zd2 ~ dbern(0.5)

zd3 ~ dbern(0.5)

zd4 ~ dbern(0.5)

ze1 ~ dbern(0.5)

a1 <-a1latent[za1+1]

a2 <-a2latent[za2+1]

a3 <-a3latent[za3+1]

b1 <-b1latent[zb1+1]

c1 <-c1latent[zc1+1]

c2 <-c2latent[zc2+1]

c3 <-c3latent[zc3+1]

c4 <-c4latent[zc4+1]

d1 <-d1latent[zd1+1]

d2 <-d2latent[zd2+1]

d3 <-d3latent[zd3+1]

d4 <-d4latent[zd4+1]

e1 <-e1latent[ze1+1]

alpha1 <-za1*a1

alpha2 <-za2*a2

alpha3 <-za3*a3

beta1 <-zb1*b1

chi1 <-zc1*c1

chi2 <-zc2*c2

chi3 <-zc3*c3

chi4 <-zc4*c4

delta1 <-zd1*d1

delta2 <-zd2*d2

delta3 <-zd3*d3

delta4 <-zd4*d4

eta1 <-ze1*e1

for (k in 1:nsites) {

#time 1

log(lambda[k]) <- a0+alpha1*dnbr[k]+alpha2*nbox[k]+alpha3*tfire[k,1]+alpha4[transect[k]]

N[1,k]~dpois(lambda[k]) #abundance

#-----point count detection-----

for (j in 1:nsess){

logit(p.p[1,k,j]) <- lb0 + beta1*nbox[k] + beta2[obs[1,k,j]] #detection

temp[1,k,j] <- 1-pow((1-p.p[1,k,j]),N[1,k])

Y.pnt[1,k,j] ~ dbern(temp[1,k,j])

#_____Bayesian GOF_________

exp_y[1,k,j] <- temp[1,k,j]

#simulated data under model

ynew[1,k,j] ~ dbern(temp[1,k,j])

#squared loss

loss_data[1,k,j] <- pow((exp_y[1,k,j]-Y.pnt[1,k,j]),2)

loss_sim[1,k,j] <- pow((exp_y[1,k,j]-ynew[1,k,j]),2)

}

#time > 1

for (t in 2:nyears){

logit(phi[t-1,k]) <- lc0 + chi1*nbox[k] + chi2*tfire[k,t] + chi3*live[t,k] + chi4*snag[t,k] #survival

S[t-1,k] ~ dbin(phi[t-1,k],N[t-1,k]) #number that survived

log(gam[t-1,k]) <- d0 + delta1*nbox[k] + delta2*tfire[k,t] + delta3*live[t,k] + delta4*snag[t,k] #reproduction

G[t-1,k] ~ dpois(gam[t-1,k])

N[t,k]<- S[t-1,k] + G[t-1,k]

#-----point count detection-----

for (j in 1:nsess){

logit(p.p[t,k,j]) <- b0 + beta1*nbox[k] + beta2[obs[t,k,j]] #detection

temp[t,k,j] <- 1-pow((1-p.p[t,k,j]),N[t,k])

Y.pnt[t,k,j] ~ dbern(temp[t,k,j])

#_____Bayesian GOF_________

exp_y[t,k,j] <- temp[t,k,j]

#simulated data under model

ynew[t,k,j] ~ dbern(temp[t,k,j])

#squared loss

loss_data[t,k,j] <- pow((exp_y[t,k,j]-Y.pnt[t,k,j]),2)

loss_sim[t,k,j] <- pow((exp_y[t,k,j]-ynew[t,k,j]),2)

}

}

}

#--------banding data-------------

for(i in 1:nind){

#detection

logit(p.b[i])<-lpb0 + eta1*sex[i]

#individuals enter sample with probability 1

Z[i,first[i]] ~ dbern(1)

for (t in 1:first[i]){

loss.data.b[i,t]<-0

loss.sim.b[i,t]<-0

}

for (t in (first[i]+1):nyears){

mu1[i,t] <- phi[t-1,loc[i]]*Z[i,t-1]

Z[i,t] ~ dbern(mu1[i,t])

mu2[i,t] <- p.b[i,t]*Z[i,t]

Y.band[i,t] ~ dbern(mu2[i,t])

#_____Bayesian GOF_________

exp_y.b[i,t] <- mu2[i,t]

#simulated data under model

ynew.b[i,t] ~ dbern(mu2[i,t])

#squared loss

loss.data.b[i,t] <- pow((exp_y.b[i,t]-Y.band[i,t]),2)

loss.sim.b[i,t] <- pow((exp_y.b[i,t]-ynew.b[i,t]),2)

}

}

#Bayesian GOF

sum_data_loss.p <-sum(loss_data[,,])

sum_sim_loss.p <-sum(loss_sim[,,])

sum.data.loss.b <-sum(loss.data.b[,])

sum.sim.loss.b <-sum(loss.sim.b[,])

sum_sim_loss <-sum_sim_loss.p + sum.sim.loss.b

sum_data_loss <-sum_data_loss.p + sum.data.loss.b

#test statistics should be ~0.5 if model fits

test<-step(sum_sim_loss-sum_data_loss)

}
